# The Extent of Interlayer Bond Strength during Fused Filament Fabrication of Nylon Copolymers: An Interplay between Thermal History and Crystalline Morphology

**DOI:** 10.3390/polym13162677

**Published:** 2021-08-11

**Authors:** Dries Vaes, Margot Coppens, Bart Goderis, Wim Zoetelief, Peter Van Puyvelde

**Affiliations:** 1 Department of Chemical Engineering, KU Leuven, Celestijnenlaan 200J Box 2424, 3001 Leuven, Belgium; dries.vaes@kuleuven.be (D.V.); margot.coppens@kuleuven.be (M.C.); 2 Department of Chemistry, KU Leuven, Celestijnenlaan 200F Box 2404, 3001 Leuven, Belgium; bart.goderis@kuleuven.be; 3 DSM Additive Manufacturing, Urmonderbaan 22, 6167 RD Geleen, The Netherlands; wim.zoetelief@dsm.com

**Keywords:** Additive Manufacturing, Fused Filament Fabrication, Fused Deposition Modeling, semi-crystalline polymers, polymer crystallization, layer adhesion, weld strength

## Abstract

One of the main drawbacks of Fused Filament Fabrication is the often-inadequate mechanical performance of printed parts due to a lack of sufficient interlayer bonding between successively deposited layers. The phenomenon of interlayer bonding becomes especially complex for semi-crystalline polymers, as, besides the extremely non-isothermal temperature history experienced by the extruded layers, the ongoing crystallization process will greatly complicate its analysis. This work attempts to elucidate a possible relation between the degree of crystallinity attained during printing by mimicking the experienced thermal history with Fast Scanning Chip Calorimetry, the extent of interlayer bonding by performing trouser tear fracture tests on printed specimens, and the resulting crystalline morphology at the weld interface through visualization with polarized light microscopy. Different printing conditions are defined, which all vary in terms of processing parameters or feedstock molecular weight. The concept of an equivalent isothermal weld time is utilized to validate whether an amorphous healing theory is capable of explaining the observed trends in weld strength. Interlayer bond strength was found to be positively impacted by an increased liquefier temperature and reduced feedstock molecular weight as predicted by the weld time. An increase in liquefier temperature of 40 °C brings about a tear energy value that is three to four times higher. The print speed was found to have a negligible effect. An elevated build plate temperature will lead to an increased degree of crystallinity, generally resulting in about a 1.5 times larger crystalline fraction compared to when printing occurs at a lower build plate temperature, as well as larger spherulites attained during printing, as it allows crystallization to occur at higher temperatures. Due to slower crystal growth, a lower tie chain density in the amorphous interlamellar regions is believed to be created, which will negatively impact interlayer bond strength.

## 1. Introduction

Fused Filament Fabrication (FFF) has become one of the most popular polymer-based production processes within the Additive Manufacturing (AM) family [1]. The technique, which is simple, highly flexible, and rather inexpensive offers a wider range of possible feedstock polymers and is less energy-consuming in comparison to the other conventional AM techniques employing polymeric feedstock, such as Stereolithography (SLA) or Selective Laser Sintering (SLS) [2,3,4]. FFF comprises the extrusion of a thermoplastic polymer filament, typically guided from a filament spool into the print head by a pinch roller mechanism. The print head consists of two parts: a heated liquefier section, where the polymer is molten, and a smaller print nozzle through which the molten polymer is extruded. During printing, filament is continuously fed into the print head, moving at a set print speed in the xy-plane, which results in the solid portion of the filament acting as a plunger so molten polymer can be deposited according to a predefined pattern onto the heated build plate, thus making up the first layer of the envisioned 3D object. The build plate will then lower by one layer height so that the extrusion process can be repeated over and over until the object is completed in a layer-wise manner [1,5,6]. Ongoing developments and improvements of AM technologies, including FFF, have sparked a clear transition over the last few years. Where these techniques were often used as means for rapid prototyping in the past, nowadays, they have evolved into stand-alone production processes suited for the fabrication of functional products and parts of high quality for more high-end and technical applications. This trend is predicted to become even more prominent in the future [7]. However, the most frequently utilized feedstock materials for FFF, namely the commodity polymers acrylonitrile butadiene styrene (ABS) and polylactic acid (PLA), which are respectively amorphous and semi-crystalline in nature, generally cannot meet the demands for these cutting-edge applications [5,8,9]. Hence, the current material range offered by FFF should be expanded through the addition of more engineering and high-performance thermoplastics, which are very often semi-crystalline polymers whose processing can become complicated by the crystallization process [7,8]. A thorough understanding of the extent of crystallization during FFF and its relation to final printed part quality will therefore become highly beneficial to successfully employ these polymers as feedstock materials in FFF for high-end applications in the future.

Upon crystallization, crystalline regions, also known as lamellae in which sections of chains are densely and orderly packed parallel to each other, are formed [10]. The semi-crystalline microstructure, consisting of these crystalline regions with amorphous regions in between, grants semi-crystalline thermoplastics increased stiffness, strength, and wear resistance compared to amorphous polymers [10,11]. In most cases, semi-crystalline polymers can be employed up to higher service temperatures while adequately retaining their mechanical properties up until their melting temperature [11,12,13]. A detrimental result of the chain packing during crystallization is that semi-crystalline polymers typically exhibit much more drastic shrinkage upon cooling than their amorphous counterparts, often giving rise to issues regarding dimensional accuracy and part distortions [12,14]. Semi-crystalline polymers generally excel in terms of chemical resistance, as well as biocompatibility, making them ideal for biomedical applications [14]. Applications utilizing semi-crystalline feedstock with FFF seek to benefit from these advantages and encompass fields, such as medicine [15,16,17,18,19], aerospace [20] and electronics [21,22,23].

Besides its many advantages, FFF generally results in limited part resolution and poor surface quality. Similarly to most other AM techniques, parts produced with FFF often suffer from unsatisfactory mechanical properties, which is mostly a direct result from inadequate fusion between subsequently extruded layers, particularly in the z-direction [2,3,4,24,25]. Especially compared to more traditional polymer processing technologies, such as compression or injection molding, FFF printed parts usually possess inferior mechanical properties, which cannot match the requirements for certain applications [26,27]. Interlayer bonding consists of diffusion and re-entanglement of polymer chains across the interlayer interface, often termed the weld zone. Macromolecular mobility at the weld zone is greatly influenced by the experienced strongly non-isothermal temperature history [25,28,29,30].

For amorphous thermoplastics, the glass transition temperature (T_g_) is considered to be the limiting temperature for macromolecular chain diffusion, as chain mobility is considered to be negligible below T_g_ [9,31]. Seppala et al. (2017) have linked macromolecular chain mobility to the inverse of the temperature-dependent shift factor a_T_(T) from time-temperature superposition on rheological data of ABS. The recorded non-isothermal temperature profile of a weld zone could then be converted into an equivalent isothermal weld time, which allows one to compare the extent of interlayer bonding between distinct sets of print settings. It was observed that the weld time could serve as a prediction for the developed strength showing that an increased liquefier temperature (T_liquefier_) will considerably improve weld strength, reflected in a higher weld time, yet print speed was found to have a negligible effect, especially at lower print speeds [30]. These findings were further substantiated in the work by Davis et al. (2017) who were the first to measure weld strength of a single weld through Mode III torsional ‘trouser tear’ tests on single-layer walls provided with a pre-crack to guide crack propagation along the weld line [32]. The effect of an increased T_liquefier_ is two-fold: it will prolong the time the weld zone temperature will stay above T_g_ and it can lead to locally remelting the previously deposited polymer at the interface, which substantially enhances diffusion and bonding [29,33]. It is generally believed that the build plate temperature (T_build plate_) will only impact molecular mobility if it is set high enough so that the printed polymer can maintain its temperature above T_g_ and hence will affect layer adhesion to a lesser degree than T_liquefier_ [33]. Some authors have made successful attempts at modeling the bond formation between successively deposited layers to predict weld strength based on thermal history and rheological data [25,34,35,36,37].

In the case of semi-crystalline feedstock materials, once deposited, the molten polymer will start cooling down, triggering crystallization. Polymer chain mobility is expected to be dramatically hindered as a direct result of the ongoing crystallization process, where macromolecular chains become part of growing crystalline regions. Hence, the establishment of adequate interlayer diffusion to ensure sufficiently strong bonds can therefore become disrupted by crystallization, making the study of the extent of weld strength for semi-crystalline polymers highly challenging [38,39,40,41,42,43,44]. Once the extruded polymer has cooled down to the crystallization onset temperature, chain interdiffusion will become limited. The weld zone temperature should therefore remain above the crystallization onset temperature for an ample amount of time [44]. It is generally believed that enhanced mechanical properties are obtained if crystallization can take place across the interlayer interface, yet for this to occur, a certain extent of interdiffusion should first ensue [31,40,45]. Similarly to their amorphous counterparts, semi-crystalline feedstock polymers benefit from being extruded at higher T_liquefier_ so that elevated weld interface temperatures can be attained, enhancing interlayer bonding [44,46,47,48,49]. An elevated build plate temperature can induce a comparable, yet less profound effect [44,47]. However, a higher T_build plate_ has been found to considerably enhance the attained degree of crystallinity and lead to thicker lamellae through an annealing effect by the heated build plate, which improves mechanical strength when loaded along the layer deposition direction [49]. Costanzo et al. (2020) have reported a significant effect of print speed (v_print_) through its impact on molecular orientation upon extrusion leading to a decrease in weld strength. Residual molecular alignment at the weld zone was found to be alleviated by increasing T_liquefier_ or decreasing v_print_ [50,51]. Furthermore, the developed crystalline morphology can impact the final interlayer bond strength. A discrepancy in spherulite size between the bulk and weld region of printed layers was observed by McIlroy et al. (2019), where smaller spherulites were found in the weld zone which could enhance mechanical strength, as a reduced spherulite size will lead to more ductile behavior [52]. Wang et al. (2017) found the welding zone to possess a higher degree of crystallinity compared to the bulk which they attribute to higher local overall temperatures. Additionally, a crystal band across the interface was observed which can positively impact the resulting interfacial bond strength [53].

This study builds further upon the investigation of the effect of printing conditions on the crystallinity developed during FFF processing of two nylon random copolymers with distinct molecular weights through mimicking the corresponding thermal profiles in a Fast Scanning Chip Calorimetry (FSC) device, as discussed in a previous study [54]. The work described here aims to elucidate a possible link between the thermal history experienced by a deposited layer, the developed degree of crystallinity, the resulting crystalline morphology in terms of spherulite size, the equivalent isothermal weld time calculated from the thermal profile, and the interlayer weld strength. Special attention is given to their respective dependence on the studied printing conditions and feedstock molecular weight, thus contributing to a better understanding of the interlayer bonding phenomenon with respect to semi-crystalline feedstock polymers for FFF.

## 2. Materials and Methods

### 2.1. Filament Feedstock and Characterization

The employed polymeric feedstock filaments, consisting of two polyamide (PA) 6/66 random copolymers, are referred to as HMWPA (high molecular weight PA) and LMWPA (low molecular weight PA), respectively, and were previously employed in earlier work [54]. Table 1 provides a summary of the main material properties as provided by the filament supplier, including the weight average molecular weight (M_w_) [kg/mol], the glass transition temperature (T_g_) [°C] and the melting temperature (T_m_) [°C] of each copolymer. It should be noted that the reported values of T_g_ and T_m_ are confirmed by Differential Scanning Calorimetry (DSC) in a DSC Q2000 (TA Instruments, New Castle, DE, USA) under a nitrogen atmosphere. During DSC analysis, the samples are subsequently heated from 20 °C to 250 °C and cooled back down to 20 °C at a rate of 10 °C/min for two cycles. Both feedstock filament spools are dried prior to use for thermal analysis, rheological measurements or FFF printing to counteract possible moisture uptake, which is typical for polyamides. Drying is performed for 24 h at 80 °C under vacuum in a Vacutherm VT 6025 vacuum drying oven (Thermo Fisher Scientific, Waltham, MA, USA). To avoid any uptake of moisture during FFF printing, the filament spools are directly placed in a PrintDry filament drying station (PrintDry, Windsor, ON, Canada), set at 70 °C and equipped with silica gel. Appendix A describes the determination of the comonomer content in the employed feedstock copolymers as knowledge of the principal constituent in the copolymers is necessary to correctly convert a melting enthalpy value obtained from thermal analysis into an absolute degree of crystallinity. A rheological characterization of the nylon feedstock is performed, which will form the basis of the calculation of the equivalent isothermal weld time, as is outlined in Appendix B.

### 2.2. Thermal History, Crystallinity and the Equivalent Isothermal Weld Time

An Ultimaker 2 FFF printer (Ultimaker, Geldermalsen, The Netherlands) is employed to execute the custom G-code that is written to print the wall geometries with an identical set-up as already described in previous work [54]. Again, a nozzle diameter of 0.4 mm is utilized, and the layer height is set to 0.2 mm. A set of nine printing conditions is defined which consists of the same six conditions previously investigated in [54], expanded with three additional ones. The printing conditions either differ in terms of the employed feedstock polymer or the applied print settings, including T_liquefier_ [°C], T_build plate_ [°C], and v_print_ [mm/s]. All examined print settings are summarized in Table 2. The extra three printing conditions, which all employ a low T_build plate_ of 40 °C, are specifically added to discern the effects of T_liquefier_ and v_print_ on crystallinity and interlayer bond strength without T_build plate_ possibly overshadowing their impact. The wall sample geometries, which are 50 layers tall and have a thickness of one single layer, are fabricated for infrared (IR) thermography and sectioning with the microtome.

IR thermography is employed to measure the temperature evolution during printing. Thermal history of the middle of layers 10 and 40 is recorded with an identical set-up as described in earlier work [54]. The temperature profiles recorded with the Optris PI 640 IR camera (Optris GmbH, Berlin, Germany) are further utilized to study the development of crystallinity with FSC and for the calculation of an equivalent isothermal weld time. The thermal history from IR thermography, as experienced by the monitored layers, can be mimicked with FSC to evaluate the attained degree of crystallinity at specific points in time during the printing process, which allows to study the effect of processing parameters on the extent of crystallization. Previous work comprises a thorough description of this developed methodology, including the conversion of IR thermal data into protocols for FSC, the preparation of sample chips, the determination of sample mass and the establishment of a correction factor to correct for possible changes occurring in the sample [54]. In this work, three separate sample chips are prepared for each employed copolymer. For each printing condition, both the full thermal history, as well as segments of it are simulated in the FSC device (Flash DSC 1, Mettler Toledo, Columbus, OH, USA). Afterwards, the sample is exposed to a heating cycle at a heating rate of 100 °C/s for which the resulting melting peak can be integrated to obtain a specific melting enthalpy ∆h_m_ [J/g]. This is performed in triplicate–once per prepared sample chip–to assess the reproducibility of the obtained data, as the average and standard deviation of each set of three melting enthalpies is calculated. Finally, the conversion of a melting enthalpy value ∆h_m_ [J/g] to an absolute degree of crystallinity X_c_ [%] can be performed using Equation (1), where ∆h_m_ [J/g] is the specific melting enthalpy obtained from thermal analysis and ∆h_100%, PA^*^_ [J/g] is the melting enthalpy for 100 % crystalline PA 6 or PA 66, which equal 230 and 255.41 J/g, respectively [55]. Here, PA* refers to the nylon comonomer which is found to be the principal constituent, as is described in Appendix A. The minority component is assumed to remain amorphous [56].

(1) $$X_{c}=\frac{\Delta h_{m}}{\Delta h_{100\%,{\mathrm{PA}}^{*}}}$$

The non-isothermal temperature profiles recorded with IR thermography are converted into equivalent isothermal weld times following the procedure developed by Seppala et al. (2017) for amorphous ABS [30]. The calculation is based on the crucial assumption that the macromolecular chain mobility and thus interlayer diffusion for the employed semi-crystalline copolymers is only dependent on temperature and is not at all influenced by the crystallization phenomenon. Hence, similarly as for amorphous polymers, chain mobility, and by extension interlayer bonding, is assumed to cease completely when the temperature of the interlayer interface equals the respective copolymer’s glass transition temperature. The weld time t_weld_ [s] calculation is performed by numerical integration of the inverse of the temperature-dependent shift factor a_T_(T), obtained by rheological measurements, as described in Appendix B, over the full recorded thermal profile. Since for each printing condition, the full temperature evolution as a function of time T(t) [°C] is known for the center of layers 10 and 40, the numerical integration is equivalent with the integration of the inverse of the time-dependent shift factor a_T_(t) with respect to time t [s] starting from the time of deposition (t = 0) up until the time where the measured temperature equals T_g_ (t = t_T = T_g__) or, for some printing conditions, until the end of the recorded temperature profile. The employed T_g_ values are given in Table 1, and do not take into account the typical shift in the glass transition to higher values due to a reduced mobility of the polymer chains as they become more anchored in the crystalline regions upon crystallization [57]. However, this will not significantly impact the calculated weld time, since the contribution of temperatures close to T_g_ will be negligible anyway. Equation (2) represents the calculation of the equivalent isothermal weld time. This calculation is applied for each recorded thermal profile thus for all nine defined printing conditions and for both monitored layer positions, that is, the middle of layers 10 and 40. Ideally, the temperature profile of the weld interface should be used for this calculation. However, due to insufficient resolution of the employed IR camera, it is impossible to optimally focus on the desired weld region. The isothermal weld time allows one to predict the extent of interlayer bonding and its dependence on processing parameters by comparing different printing conditions, solely based on the thermal history experienced by the deposited polymer layers. Hence, it can be employed to check whether amorphous healing theory is able to explain the observed trends in terms of interlayer bond strength. Physically, the equivalent isothermal weld time can be interpreted as follows: taking a reference temperature of 230 °C for HMWPA to obtain the shift factors from time-temperature superposition, an equivalent isothermal weld time of 1 s calculated from a complete thermal profile then signifies that the extent of chain mobility, and thus, interlayer diffusion at the interface over the full thermal profile is equal to that when holding the interface isothermally for 1 s at 230 °C.

(2) $$t_{weld}=\int_{0}^{t_{T=T_{g}}}\frac{dt}{a_{T}\left(t\right)}$$

### 2.3. Fracture Tests

**Figure 1:**
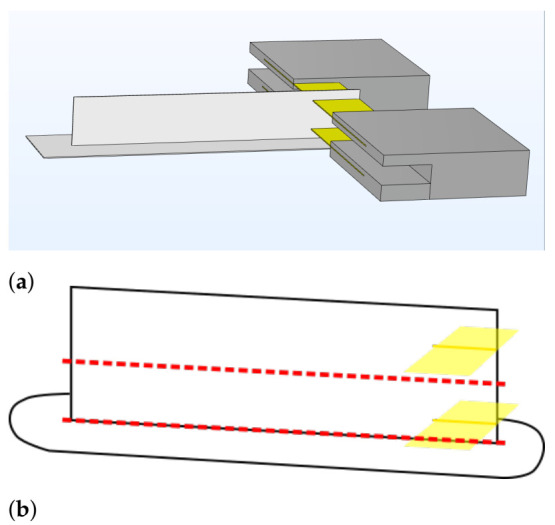
A schematic representation of the methodology to manufacture fracture test samples with (**a**) the wall geometry and brim (white), the PC Kapton tape holders (grey) and the strips of double-sided Kapton tape inserted to create pre-cracks (yellow) and (**b**) the printed wall specimen with Kapton tape strips (yellow) with the red dotted lines indicating where the sample will be cut to obtain two separate samples to test interlayer bond strength.

In order to test interlayer bond strength, wall geometries are manufactured according to an adapted version of the methodology employed by Davis et al. (2017) [32]. During printing, a 1 cm wide piece of double-sided Kapton tape is placed at the edge of the printed wall specimens right at the interface between layers 9 and 10 and between layers 39 and 40 to create a pre-crack at these specific interlayer weld regions. This pre-crack will direct the crack propagation along the weld interface during mechanical testing. Figure 1a illustrates the working principle. The strips of Kapton tape are held in place by custom-designed holders printed out of polycarbonate (PC) filament. The pre-cracks are created by sliding each respective Kapton tape holder over the corresponding weld interface at the edge of the wall at the correct timepoint during printing. The formation of pre-cracks can thus be performed with great reproducibility and uniformity, as the Kapton tape holders ensure that the tape is placed at exactly the correct height each time. Each printed wall specimen thus consists of two separate samples which can be employed to test the interlayer bond strength of each respective interface. To obtain the two samples, the wall specimen is cut horizontally along the middle and the brim is removed, as is depicted by the red dotted lines on Figure 1b. For each of the nine printing conditions, five wall specimens are printed.

**Figure 2:**
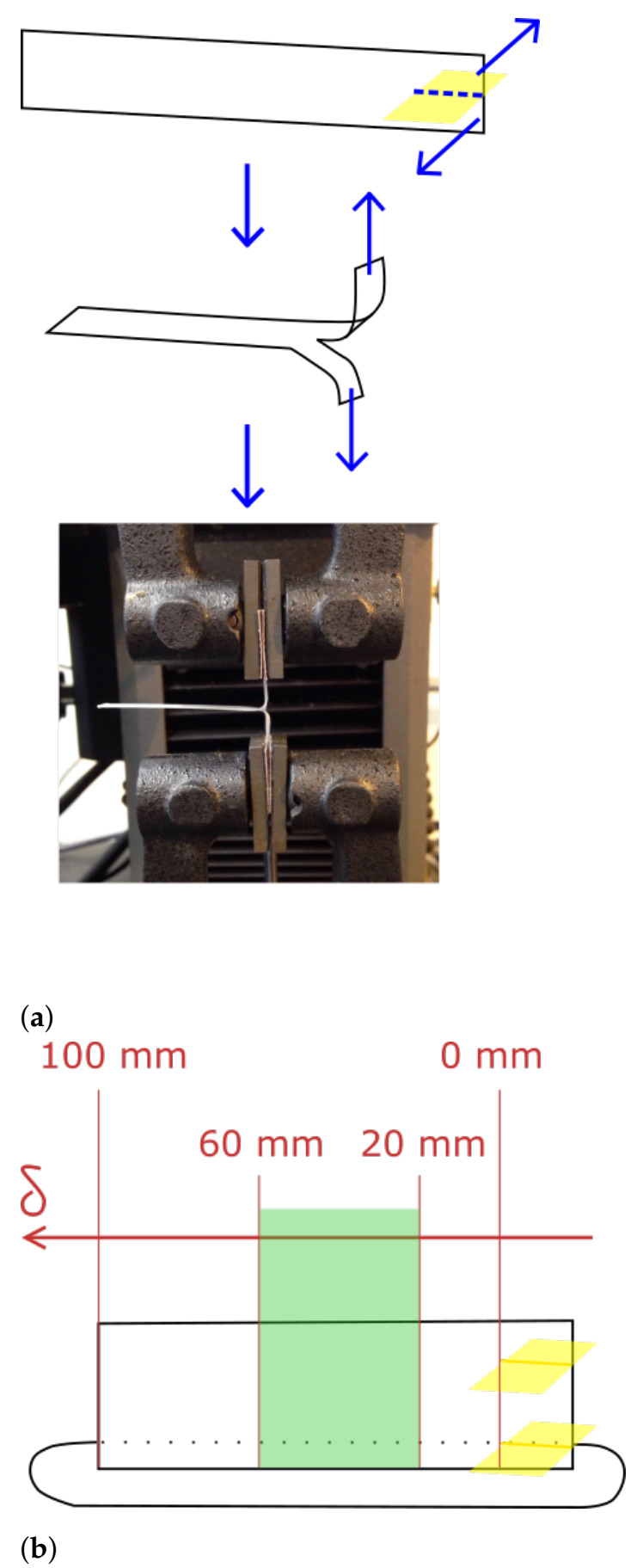
The steps in the employed fracture test methodology with (**a**) loading a specimen for a Mode III fracture test consisting of removing the Kapton tape insert to reveal the pre-crack, followed by clamping the ends on either side of the pre-crack in the pneumatic grips of the tensile tester together with sandpaper, and (**b**) a schematic representation of the region of interest of the wall specimen (green) corresponding to the load data between displacements of 20 and 60 mm, which will be used for data analysis.

Mode III fracture tests, or so-called ‘trouser tear’ tests, are executed on an Instron 5943 tensile tester (Instron, Norwood, MA, USA) equipped with a load cell of 100 N. Before sample insertion, the gauge length is set to zero and the load is balanced. Figure 2a depicts the steps followed to load the sample for the mechanical test. Initially, the Kapton tape insert is removed to obtain a pre-crack. The ends on either side of the pre-crack are then clamped between the 6 bar pneumatic grips of the tensile tester. Sandpaper is placed around the clamped ends to prevent slip during testing. The fracture test starts by applying an extension of the upper grip at a rate of 1 mm/min until a pre-load of 0.2 N is achieved to alleviate possible compressive forces applied upon loading which will improve reproducibility. Afterwards, the actual trouser tear test initiates at a rate of 1 mm/s while measuring the load F [N] required to pull the interlayer interface apart as a function of the displacement δ [mm] until the sample is completely torn apart. The total displacement will be approximately 100 mm, that is, the double of the remaining interlayer surface length which is equal to the wall length of 60 mm minus the pre-crack length of 10 mm. To focus on the middle section of the wall and to exclude effects of the wall edges, the average of the load data between displacements of 20 and 60 mm is taken. This displacement range of 40 mm, shown in green in Figure 2b, which corresponds to the central 20 mm of interlayer interface, is chosen as a trade-off between minimizing variance and avoiding edge effects. The trouser tear test is performed on ten samples for each printing condition. The measured load data for each specimen in the displacement range between 20 and 60 mm corresponds to a set of 2000 data points of which the average and standard deviation are calculated. For each set of five samples, corresponding to one specific interlayer interface for a single printing condition, an average load is calculated based on the average values obtained for each separate specimen. The error on the data is expressed as a pooled standard deviation.

### 2.4. Sectioning and Visualization

**Figure 3:**
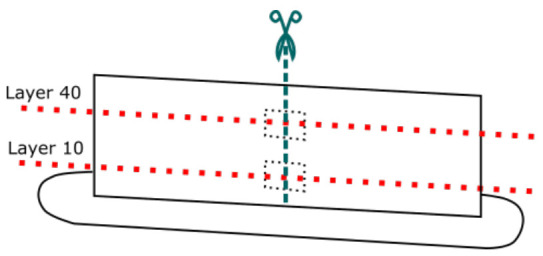
A schematic depiction of the methodology followed for sectioning of the printed wall geometries by cutting out rectangular specimens around the middle regions of layers 10 and 40, which will be subsequently sectioned perpendicular to the layer direction in the microtome.

A Leica Ultracut UCT microtome (Leica, Wetzlar, Germany), equipped with a Leica EM FCS low temperature sectioning system with both a glass and diamond knife, is utilized for sectioning of the printed wall geometries, one for each printing condition. Sectioning is performed under ambient conditions. Rectangular specimens are cut out around the middle region of layers 10 and 40 for each wall geometry. These rectangular specimens are then clamped in the microtome for further sectioning perpendicular to the layer direction. This employed methodology is illustrated schematically in Figure 3. Initially, a glass knife (LKB-Produkter AB, Bromma, Sweden) is utilized to coarsely remove part of the top surface of the clamped specimen at a rate of 1 mm/s in about 40 steps of 10 μm. Afterwards, a cryo diamond microtome knife (Diatome, Nidau, Switzerland) with a knife angle of 35° and a size of 3 mm is employed for finer sectioning at a rate of 1 mm/s of the remaining specimen resulting in sections with a thickness of 10 μm which are collected in an oil bath. The sections are removed from the oil bath with a Diatome Perfect Loop tool (Diatome, Nidau, Switzerland) and subsequently placed between glass microscopy slides to be visualized with Polarized Light Microscopy (PLM). The residual specimen clamped in the microtome will have obtained a smoothened surface which can be analyzed under a stereomicroscope for the determination of the contact widths, often called weld lengths, between the successively deposited layers. Sectioning in the microtome thus fulfills a two-fold purpose, providing samples for both weld length determination, as well as morphology observation.

**Figure 4:**
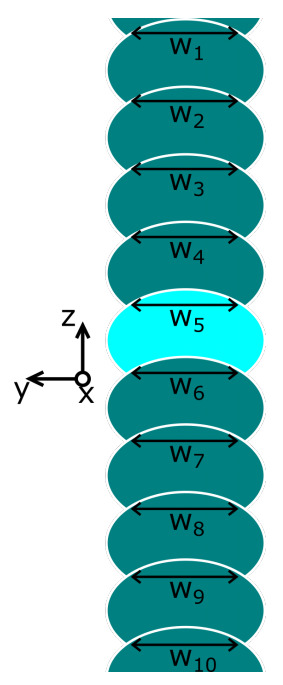
A schematic illustration of the determination of the weld lengths around the layer of interest (layer 10 or 40), which is highlighted. Each ellipsoid thus represents a cross-section of a layer perpendicular to the layer direction.

The contact width at the interlayer interface, typically termed weld length w [μm], between successively deposited layers is determined for ten interlayer interfaces around the layers of interest that is, layers 10 and 40, for each printing condition. Figure 4 shows a schematic depiction of the concept of weld length measurements. The sample with a smooth cross-section obtained by sectioning with the microtome will be placed under a Keyence Digital Microscope VHX-600 (Keyence, Osaka, Japan), equipped with a Dual Objective Zoom Lens VH-ZST, providing a 300x magnification. Full ring lighting is applied. To obtain a clear picture of the full cross-section of the sample, HDR 3D stitching is employed. Weld lengths of the ten separate interlayer contact interfaces are then measured by manual two-point measurements on the acquired image. For each set of ten measured weld lengths, the average and standard error are calculated.

As interlayer bond strength is directly dependent on the width of the interlayer contact interface, the average loads F [N] obtained from the Mode III fracture tests are normalized by the corresponding average weld lengths w [μm] to obtain a tear energy G_III_ [kJ/m^2^]. The calculation of the tear energy is given by Equation (3) and assumes that the weld length is constant for each interlayer interface under consideration, thus excluding the error on the measured weld lengths. The tear energy provides a direct measure of the interlayer bond strength for a specific interlayer weld region, taking into account variations in weld length.

(3) $$G_{III}=\frac{F}{w}$$

The sections with thickness of 10 μm from sectioning with the microtome, which are placed between glass microscopy slides are visualized with Polarized Light Microscopy (PLM). An Olympus BX41 Phase Contrast & Darkfield Microscope (Olympus, Shinjuku, Tokyo, Japan) equipped with Olympus SLMPlan 20× and 50× objectives (Olympus, Shinjuku, Tokyo, Japan) and a Hamamatsu C4742-95 Orca 100 CCD Monochrome Camera (Hamamatsu, Hamamatsu City, Japan) is employed. Cross-polarizers are utilized to reveal Maltese crosses corresponding to spherulites of the crystalline morphology. Images are taken with the HiPic acquisition software (Hamamatsu, Hamamatsu City, Japan) around the weld regions of interest. Spherulite diameters are measured with ImageJ image analysis software. The diameter of ten spherulites around the interlayer interface is measured. An average and standard deviation are calculated for each set of ten measured spherulite diameters.

## 3. Results and Discussion

### 3.1. Thermal History

**Figure 5:**
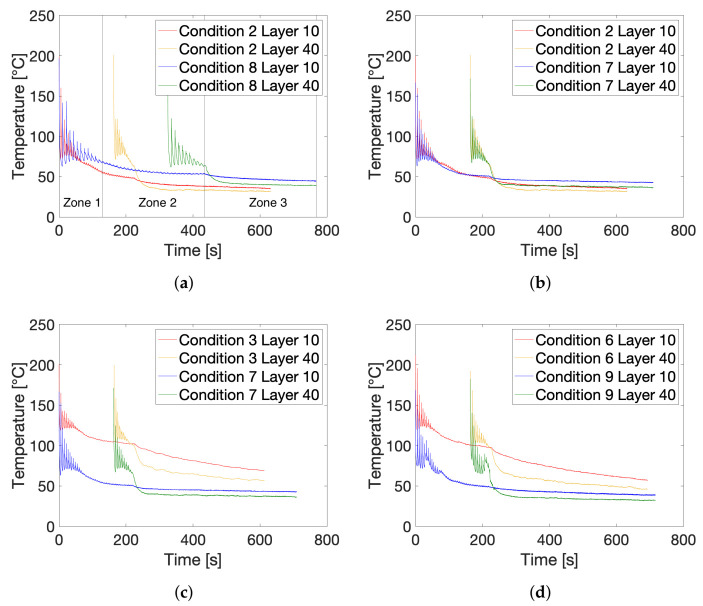
A comparison of the thermal history recorded through IR thermography of printing conditions 7 to 9 with T_build plate_ of 40 °C with respect to their counterparts printed with a build plate temperature of 110 °C that is, printing conditions 2, 3, and 6. (**a**) Comparison between v_print_ of 5.5 (Condition 8) and 11 mm/s (Condition 2) for T_build plate_ = 40 °C. (**b**) Comparison between T_liquefier_ of 260 (Condition 2) and 240 °C (Condition 7) for T_build plate_ = 40 °C. (**c**) Comparison between T_build plate_ of 40 (Condition 7) and 110 °C (Condition 3) for T_liquefier_ = 240 °C. (**d**) Comparison between T_build plate_ of 40 (Condition 9) and 110 °C (Condition 6) for LMWPA feedstock.

Figure 5 summarizes the temperature profiles experienced by layers 10 and 40 for some of the employed printing conditions obtained through IR thermography during FFF processing. A comparison is made between the temperature profiles related to different printing conditions in order to elucidate possible effects of printing parameters on the experienced temperature profiles. It should be noted that the observations made in earlier work also hold for the thermal profiles presented here [54]. Each temperature profile can be seen as a composition of three zones, of which the end points are indicated by the vertical lines for the thermal profile of the center of layer 10 for condition 8 on Figure 5a. In the initial stage of printing, corresponding to zone 1, strong variations in the experienced temperature can be observed due to newly extruded layers being printed on top of the monitored layer, which will result in cyclic heating and cooling at significant rates. The impact of these successively extruded layers dies down after a certain number of layers has been printed, which is when the second zone of the thermal history is entered, and the sharp temperature peaks are no longer recorded. As printing is still ongoing in zone 2, the layer temperature will typically decrease slowly to the set T_build plate_. Finally, when printing is finished, the monitored layer cools down even further as the build plate is now cooling down as well, which corresponds to zone 3 in the recorded temperature profiles. Since layer 40 is extruded later during FFF printing of the wall geometries, zone 2 in the thermal history, as well as the total residence time on the build plate will be much shorter for these layers.

Figure 5a compares the thermal profiles of conditions 2 and 8, which only differ in terms of the applied print speed. Clearly, by halving the print speed from 11 mm/s to 5.5 mm/s, the total build time will be doubled. As the interlayer deposition time will double as well at v_print_ of 5.5 mm/s, the time between the recorded temperature peaks will be twice as long, providing more time for the monitored layer to cool down in between successive depositions, which is reflected in the reduced lower limits of the temperature peaks for condition 8 as compared to those of condition 2. Furthermore, higher maximum temperatures of the successive peaks are measured for layers printed at lower v_print_, which might be attributed to a prolonged residence time in the liquefier resulting in improved heat transfer to the melted feedstock filament.

The effect of the liquefier temperature is illustrated by Figure 5b, comparing the thermal history of condition 2 with T_liquefier_ of 260 °C to that of condition 7 with T_liquefier_ of 240 °C, yet its effect is only visible on the initial maximum peak temperatures which are clearly higher for an elevated T_liquefier_. Both temperature profiles further follow a highly similar course after the initial peaks due to deposition of new layers become less significant.

Figure 5c,d exhibit the comparison of conditions 7 and 9, both printed at T_build plate_ of 40 °C, to their counterpart printed at a higher build plate temperature of 110 °C, namely conditions 3 and 6, respectively. The considerable impact of T_build plate_ on the experienced thermal history is apparent as the overall temperature profiles are shifted to lower temperatures when applying a T_build plate_ of 40 °C. A higher build plate temperature will increase the average temperature of the monitored layers over the course of printing which can either be attributed to conduction by the build plate itself or by raising the ambient air temperature which can then heat up the printed part through convective heat transfer, or due to a combination of both. It should be noted that the difference in thermal history between layers 10 and 40, especially after printing is completed, is more significant when printing at higher T_build plate_ as it will lead to stronger thermal gradients in the printed wall, again due to inhomogeneous conduction or convective heat transfer induced by the build plate.

### 3.2. Crystallinity

From the determination of the comonomer content described in Appendix A, it has become obvious that PA 6 is the principal constituent in the employed copolymers. Thus, Equation (1) and a value of 230 J/g for ∆h_100%, PA 6_ can be utilized to convert a melting enthalpy value obtained from thermal analysis to an absolute crystalline fraction X_c_. Hence, the degree of crystallinity is calculated based on the assumption of complete exclusion of the PA 66 comonomer from the crystalline phase so that only the PA 6 segments take part in crystallization [13,56].

**Figure 6:**
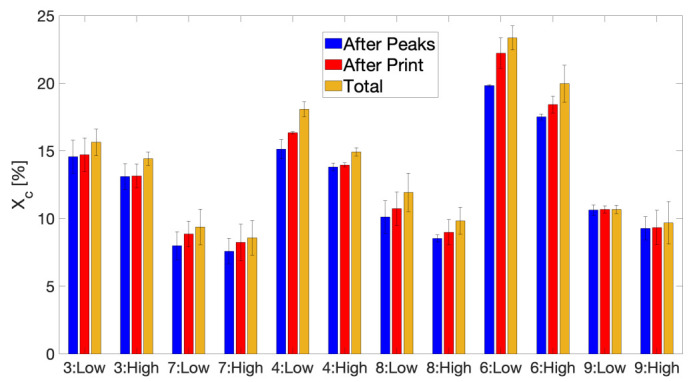
The comparison of the degrees of crystallinity X_c_ after each segment of the thermal history as obtained by FSC for conditions 3, 4 and 6 printed at T_build plate_ of 110 °C and their respective counterparts, namely conditions 7, 8 and 9, printed at T_build plate_ of 40 °C. ‘Low’ and ‘High’ refer to layers 10 and 40, respectively.

After approximation of the recorded temperature profiles by linear segments of constant heating and cooling rate, the approximated thermal history for each printing condition and respective layer position can be mimicked as a thermal protocol in the FSC device. Both the full approximated thermal profile up until the end of zone 3 (‘Total’), as well as segments of it, namely up until the initial peaks are not visible anymore at the end of zone 1 (‘After Peaks’) and up until the printing has finished at the end of zone 2 (‘After Print’), are simulated by FSC. Measurements are performed in triplicate that is, once per prepared sample chip. For conditions 1 to 6, the resulting crystalline fractions after each segment of their thermal history are reported in Appendix C with error bars to indicate reproducibility, as for these conditions the effect of processing parameters on their crystallization behavior has already been extensively described in previous work, where it became apparent that a higher build plate temperature and a lower feedstock molecular weight significantly enhance the extent of crystallization [54]. ‘Low’ and ‘High’ refer to layer 10 and layer 40, respectively. Figure 6 again illustrates the considerable impact of T_build plate_ on the attained crystallinity over the course of FFF printing by comparing each printing condition with a build plate temperature of 110 °C to its respective counterpart printed at lower T_build plate_ of 40 °C. It should be noted that, for all observed printing conditions, the vast majority of the total attained crystallinity has already been achieved after the initial peaks, associated with strong temperature fluctuations at high heating and cooling rates, in the experienced thermal history. Through heating of the printed wall geometry by the build plate, either by direct conduction or by convective heating from the surrounding air, the overall average temperature of parts printed at T_build plate_ of 110 °C is much higher compared to that for conditions printed with T_build plate_ of 40 °C. The higher T_build plate_ will allow the extruded polymer to remain at an elevated temperature within the crystallization regime throughout the printing process. Hence, crystallization takes place over a prolonged time which will strongly increase the total degree of crystallinity obtained after printing at high T_build plate_. On the contrary, for conditions printed at lower build plate temperature of 40 °C, deposited layers cool down to a temperature close to T_g_ quite early in the printing process so that crystallization will not proceed as extensively.

**Figure 7:**
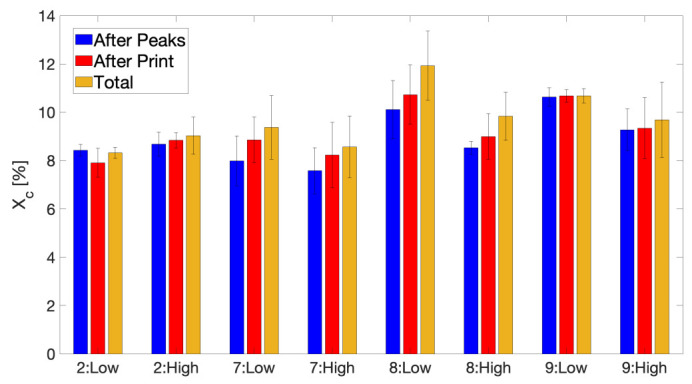
The comparison of the degrees of crystallinity X_c_ after each segment of the thermal history as obtained by FSC for conditions 2, 7, 8 and 9, all printed at low T_build plate_ of 40 °C. ‘Low’ and ‘High’ refer to layer 10 and 40, respectively.

Since the build plate temperature exerts significant influence on the crystallization phenomenon during FFF printing, the effect of other processing parameters might become overshadowed by T_build plate_. Therefore, a comparison can be made between all printing conditions with T_build plate_ set to a lower value of 40 °C to possibly discern the impact of T_liquefier_ and v_print_ on the extent of crystallization. Figure 7 provides the comparison between conditions 2, 7, 8 and 9, all fabricated with T_build plate_ of 40 °C. Even at a low build plate temperature, T_liquefier_ does not seem to impact the attained degree of crystallization, since an elevated T_liquefier_ will only increase the initial peak temperatures experienced by the deposited polymer. A lower molecular weight of the feedstock copolymer dramatically enhances crystallizability when processed at high T_build plate_, yet this effect is far less apparent for a build plate temperature of 40 °C, indicating that an elevated T_build plate_ of 110 °C is required for the LMWPA feedstock material to fully exhibit its crystallization potential. In terms of the influence of print speed, a moderately larger degree of crystallinity is achieved for lower v_print_, especially for layer 10. This can be attributed to the longer time the heated print head resides over the extruded layer at a specific location for slower print speeds, thus slightly promoting crystallization [42].

### 3.3. Weld Time as a Predictive Tool for Interlayer Bond Strength

**Figure 8:**
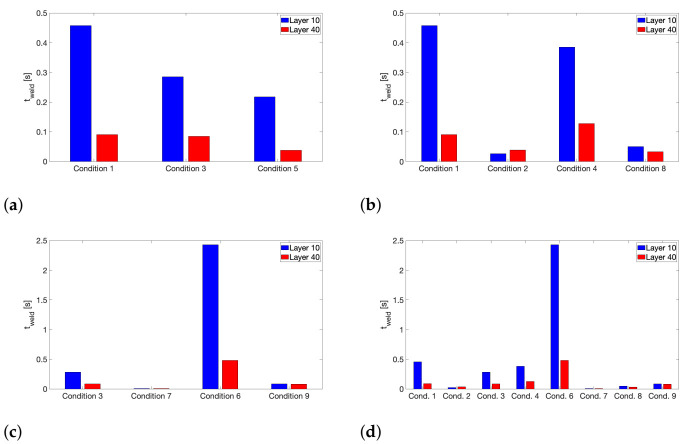
An overview of the calculated equivalent isothermal weld times t_weld_ for all printing conditions. (**a**) Effect of T_liquefier_ = 260, 240 and 220 °C for conditions 1, 3 and 5, respectively. (**b**) Effect of v_print_ = 11 mm/s for conditions 1 and 2, and 5.5 mm/s for conditions 4 and 8. (**c**) Effect of M_w_. HMWPA for conditions 3 and 7 and LMWPA for conditions 6 and 9. (**d**) Effect of T_build plate_ = 110 °C for conditions 1, 3, 4 and 6, and 40 °C for conditions 2, 7, 8 and 9.

An overview of the calculated equivalent isothermal weld times is provided in Figure 8, where special attention is given to highlight the impact of each varied processing parameter, as well as the feedstock’s molecular weight on t_weld_. Generally, the weld time based on the thermal history experienced by layer 10 will be larger than that for layer 40 printed higher above the build plate, as the latter will be printed later over the course of the deposition process. This discrepancy between the calculated weld times of layer 10 and 40 becomes especially apparent for printing conditions with elevated T_build plate_, since for these conditions, a stronger thermal gradient exists across the printed wall geometry, allowing layer 10, deposited close to the heated build plate, to remain at elevated average temperature for a prolonged time.

Figure 8a clearly illustrates the effect of T_liquefier_, where the weld time decreases monotonically with decreasing liquefier temperature, as was already observed for amorphous polymers, such as ABS [30] or for semi-crystalline polymers, such as PA 12 [49]. Higher liquefier temperatures will result in increased peak temperatures experienced by the deposited layer which will strongly benefit interlayer diffusion as is reflected by an increased t_weld_.

No significant effect of v_print_ on the weld time can be distinguished from Figure 8b. Although the total printing time is doubled when print speed is halved and thus more heat exposure can occur as a result, the largest contribution to the weld time will be originating from the initial temperature peaks in the experienced thermal history, so that a prolonged build time will not substantially affect the calculated t_weld_. Furthermore, a slower v_print_ is known to increase the time in between experienced temperature peaks which will lead to more significant cooling in between each deposition of a new layer on top of the monitored layer, yet the recorded peak temperatures are slightly higher for lower v_print_ which has previously been attributed to an extended residence time in the heated liquefier. Hence, both effects most probably cancel each other out, which might explain the absence of a visible trend in the effect of v_print_ on the weld time, as the print speeds chosen in this study are anyway rather low. Other authors have employed significantly higher print speeds, yet only noticed a very limited decrease in t_weld_ with increasing v_print_, which was far less conspicuous than the impact of T_liquefier_ [30].

**Figure A5:**
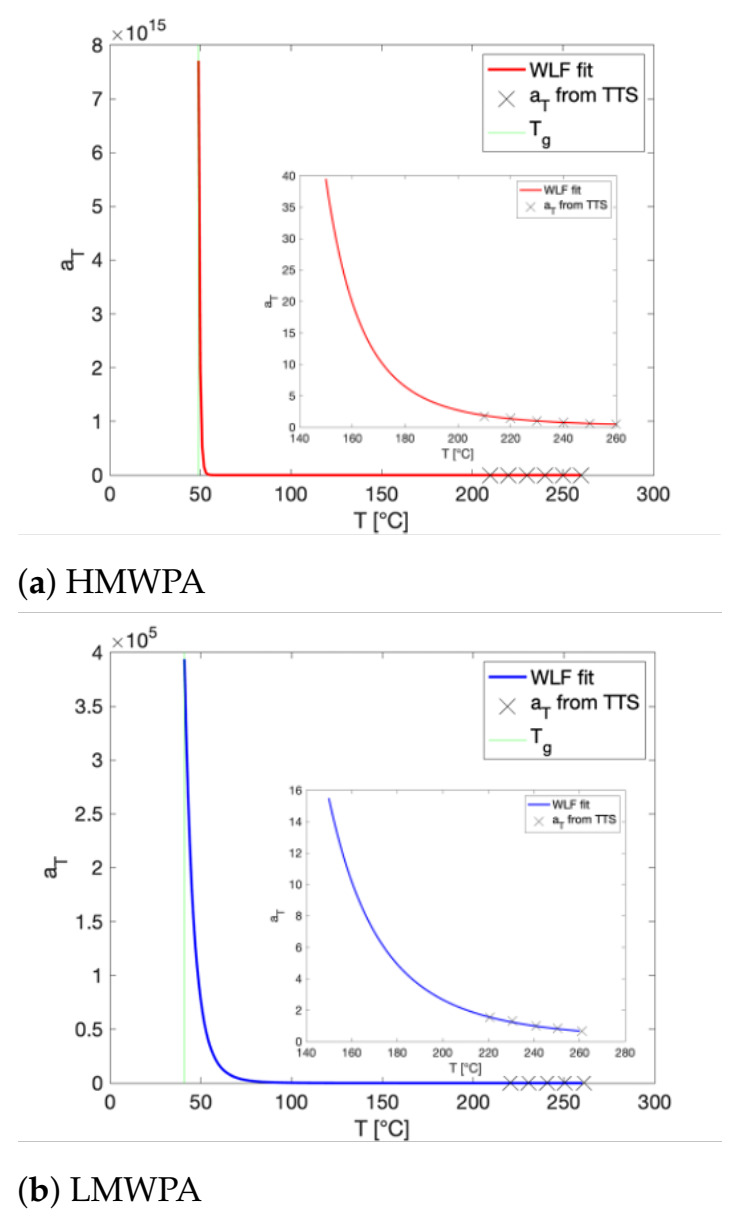
The shift factors a_T_ obtained from time-temperature superposition (TTS) on rheological data measured in the melt state with their WLF fit to extrapolate their temperature dependence up until T_g_ for (**a**) HMWPA and (**b**) LMWPA.

As the chain self-diffusion coefficient in an entangled polymer melt generally is believed to be inversely proportional to M_w_^2^ [58], a strong influence of the feedstock polymer’s molecular weight can be expected on the equivalent isothermal weld time, as is embodied by Figure 8c. Note that the impact of molecular weight on chain mobility directly manifests itself in terms of the difference in shift factors between both copolymers, calculated from time-temperature superposition on rheological data as illustrated by Figure A5 in Appendix B. The values for t_weld_ for printing conditions using LMWPA are substantially larger than for their counterparts employing HMWPA feedstock filament. Especially the weld time for layer 10 of condition 6 clearly stands out above the rest, which is a strong indication of highly enhanced interlayer diffusion and bonding.

The significance of T_build plate_ in raising the average overall temperature experienced by the deposited layers to much higher temperatures compared to those experienced by the extrudate after deposition on a build plate set at 40 °C definitely emanates from Figure 8d, where printing conditions which employ a build plate temperature of 110 °C achieve considerably higher weld times.

**Figure 9:**
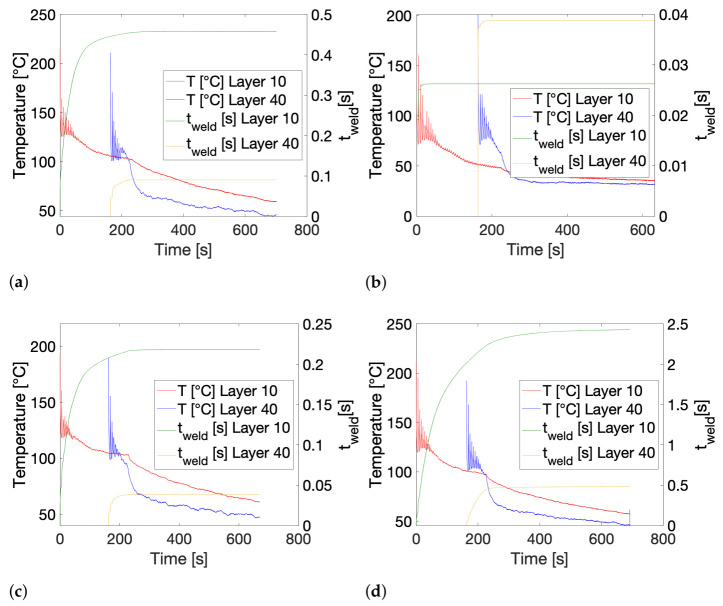
The cumulative progression of the calculated isothermal weld time versus the corresponding thermal history. (**a**) Condition 1: HMWPA, T_liquefier_ = 260 °C, v_print_ = 11 mm/s and T_build plate_ = 110 °C. (**b**) Condition 2: HMWPA, T_liquefier_ = 260 °C, v_print_ = 11 mm/s and T_build plate_ = 40 °C. (**c**) Condition 5: HMWPA, T_liquefier_ = 220 °C, v_print_ = 11 mm/s and T_build plate_ = 110 °C. (**d**) Condition 6: LMWPA, T_liquefier_ = 240 °C, v_print_ = 11 mm/s and T_build plate_ = 110 °C.

To illustrate the gradual progression in the calculated weld time through integration of the inverse shift factors over time, cumulative weld time plots are depicted in Figure 9 and compared to their corresponding temperature profiles for printing conditions 1, 2, 5 and 6. It can clearly be observed that the largest portion of the total equivalent isothermal weld time for each printing condition and layer position has already been attained after the initial temperature fluctuations in the experienced thermal history. For condition 1, shown in Figure 9a, a further, albeit limited increase in t_weld_ can be recognized after the initial temperature peaks, especially for layer 10. Once printing has finished and the printed part begins to cool down together with the build plate, t_weld_ seems to have stabilized, indicating that, during this stage in the thermal history, temperatures have already become too low to significantly impact the weld time value. The calculated weld time for condition 5, printed with a lower T_liquefier_ compared to condition 1, follows a highly similar course, as is portrayed in Figure 9c, yet acquires a noticeably lower t_weld_ as it experiences less intense peak temperatures. As the overall temperature for conditions printed with a lower T_build plate_ of 40 °C will remain rather low throughout the printing process, the weld time calculation already achieves a maximum value after the first couple of initial peaks in the thermal history for these conditions, which is illustrated by Figure 9b. The enhanced chain mobility for the LMWPA copolymer most definitely becomes evident in Figure 9d, where, especially for layer 10, even after a strong initial increase during the temperature peaks, the calculated weld time persistently increases across the full printing time. This is obviously far less predominant for layer 40 as the zone after the initial peaks only lasts for a very short time for this layer position.

### 3.4. Interlayer Bond Strength

For each printing condition the interlayer bond strength of two separate layer interfaces is studied. The interlayer weld regions under consideration are the interface between layers 9 and 10 and between layers 39 and 40, referred to as Layer 10 and Layer 40, respectively, in all further data representations. Appendixes Appendix D and Appendix E provide additional information concerning the measured loads and weld lengths, respectively.

**Figure 10:**
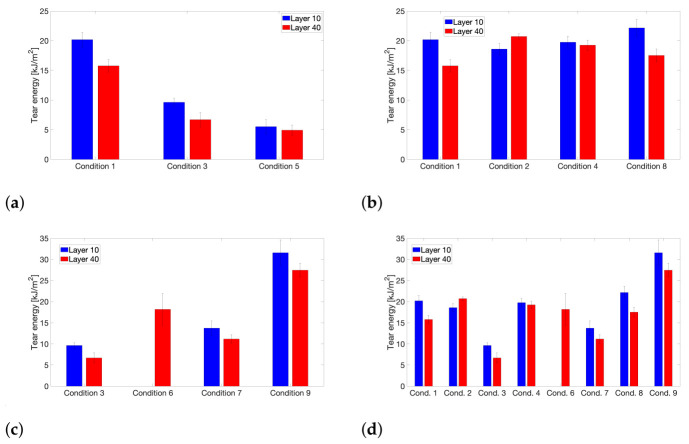
An overview of the calculated tear energies G_III_ and thus the extent of interlayer bond strength for all printing conditions. (**a**) Effect of T_liquefier_ = 260, 240 and 220 °C for conditions 1, 3 and 5, respectively. (**b**) Effect of v_print_ = 11 mm/s for conditions 1 and 2, and 5.5 mm/s for conditions 4 and 8. (**c**) Effect of M_w_. HMWPA for conditions 3 and 7 and LMWPA for conditions 6 and 9. (**d**) Effect of T_build plate_ = 110 °C for conditions 1, 3, 4 and 6, and 40 °C for conditions 2, 7, 8 and 9.

An overview of the calculated tear energies G_III_ for each printing condition and respective interlayer interface is provided in Figure 10. In general, it can be observed that the tear energy for the interlayer interface between layers 9 and 10 is slightly higher than that for the weld region between layers 39 and 40, which was already reflected in the calculated weld times, portrayed in Figure 8. This is inherent to the FFF printing process, where the existence of a thermal gradient across the printed sample will result in an inhomogeneous thermal history and thus non-uniform weld strength across the printed part [27,59].

The impact of T_liquefier_ on the tear energy is depicted in Figure 10a, showing a monotonically decreasing tear energy and thus interlayer bond strength with decreasing liquefier temperature. This is a trend that has already been extensively reported in the literature, both for amorphous feedstock, such as ABS [30,32], as well as for semi-crystalline filaments including PLA [48,60,61,62], PP [27,63,64] and PA [46,49,65]. When comparing Figure 8a and Figure 10a, both graphs exhibit a highly similar course, meaning that the calculated equivalent isothermal weld time clearly is able to predict the impact of the liquefier temperature on the resulting weld strength. A higher t_weld_ will be achieved for elevated liquefier temperatures as the experienced peak temperatures will be considerably higher, which will enhance chain mobility and macromolecular diffusion across the interface. Additionally, a higher T_liquefier_ can possibly induce partial remelting of the weld region, which will strongly benefit adhesion. It should be noted that printing conditions 1, 3 and 5, which all only differ in terms of T_liquefier_, exhibit almost identical degrees of crystallinity after FFF processing, indicating that the observed impact of T_liquefier_ purely originates from its influence on the thermal history experienced by the deposited layers at the interlayer interface. A comparison of the tear energy values of condition 2 and 7 in Figure 10d again illustrates the positive influence of T_liquefier_ on the obtained weld strength.

Similarly as for the calculated t_weld_, Figure 10b does not show a clear effect of the print speed on the interlayer bond strength. The reasons behind the absence of an evident impact of v_print_ on the reported tear energy values are identical to those given in the discussion concerning its effect on weld time. However, Costanzo et al. have reported a distinct decreasing weld strength with increasing print speed, varying v_print_ from 20 to 120 mm/s. The negative impact on bond strength can be attributed to the residual alignment due to orientation of polymer chains upon extrusion from the print nozzle. If the residual alignment cannot relax prior to solidification, insufficient entanglements can be formed leading to poorer interlayer adhesion [50,51]. The print speeds employed in this study are most likely too low to actually induce a significant change in interlayer bond strength through alignment effects upon extrusion.

**Figure A7:**
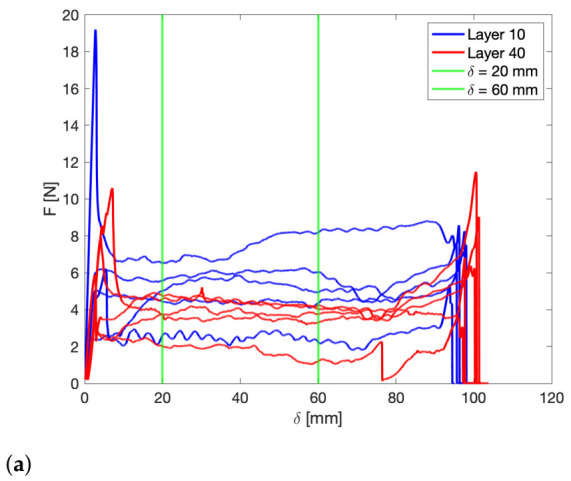

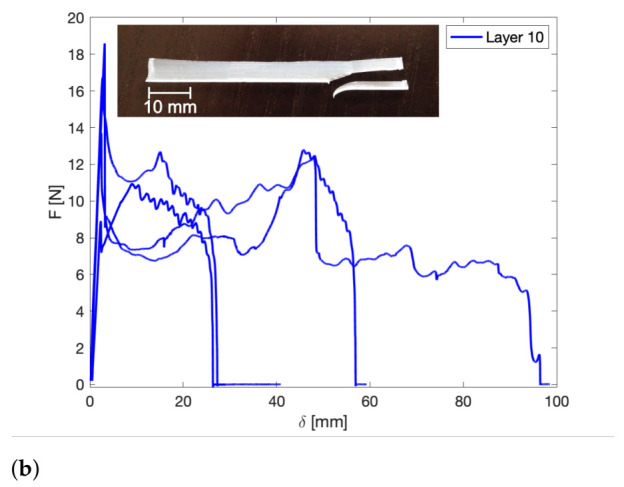
Raw data of load F [N] versus displacement δ [mm]. (**a**) Both studied weld interfaces for Condition 1: HMWPA, T_liquefier_ = 260 °C, v_print_ = 11 mm/s and T_build plate_ = 110 °C. (**b**) The weld interface between layers 9 and 10 for Condition 6: LMWPA, T_liquefier_ = 240 °C, v_print_ = 11 mm/s and T_build plate_ = 110 °C.

The enhanced molecular diffusion for the LMWPA feedstock copolymer becomes apparent when comparing conditions 3 and 6 or 7 and 9, as depicted in Figure 10c. This was again already predicted by the weld time calculation. For an identical thermal history, the LMWPA feedstock material will possess improved molecular mobility allowing for a prolonged extent of interlayer diffusion and thus increased weld strength. Other authors have also reported improved bond formation when processing filament feedstock with lower molecular weight [42,66]. Although the LMWPA copolymer exhibits enhanced crystallizability, reflected in a higher degree of crystallinity post-printing compared to the HMWPA feedstock counterpart, the effect of molecular weight on macromolecular diffusion and interlayer bonding clearly is predominant and is not negatively impacted by the ongoing crystallization process. It should be noted that for the interface between layers 9 and 10 for condition 6 no tear energy value is reported as it was impossible to obtain load data for these samples, since they failed across the layers instead of along the weld line, indicating the formation of a bulk specimen, as is illustrated in Figure A7b in Appendix D. This was already somewhat predicted by the dramatically higher value of t_weld_ that was obtained for this layer position and printing condition.

An elevated build plate temperature is generally believed to shift the experienced thermal history to higher temperatures, which will promote interlayer diffusion and result in enhanced interlayer bond strength [53,67]. This is reflected in the higher overall temperatures in the temperature profiles for printing conditions employing a higher T_build plate_ of 110 °C and in the increased values for t_weld_ for these conditions. However, Figure 10d clearly displays an opposite trend, where the tear energy values for printing conditions with a lower build plate temperature of 40 °C indicate highly similar or even improved interlayer bond strength compared to the counterparts printed at elevated T_build plate_. This is counterintuitive, since an analysis of thermal history alone can now no longer explain the obtained weld strength values. Yet, the build plate temperature has been found to considerably impact the achieved degree of crystallinity during FFF processing. Wang et al. (2018) have attributed the poorer interlayer adhesion when printing PLA at elevated T_build plate_ to the larger extent of crystallization-induced shrinkage as a direct result of the enhanced degree of crystallinity associated with higher build plate temperatures [68]. The extent and impact of shrinkage on interlayer welding is not considered in this study, although it becomes apparent that the impact of T_build plate_ on interlayer bonding is most likely directly linked to the crystallization phenomenon.

It has become clear from the analysis of the tear energies that the concept of an equivalent isothermal weld time can be utilized to predict the effect of T_liquefier_ and molecular weight on the extent of interlayer bonding, as well as the apparent lack of impact of v_print_ on interfacial adhesion, indicating that amorphous healing theory seems to be adequate to describe and explain these observations. Yet, the results concerning the effect of T_build plate_ on weld strength did not follow the weld time predictions. This discrepancy can most likely be attributed to the enhanced crystallization process at elevated build plate temperature. More extensive crystallization is expected to affect interlayer bonding by limiting macromolecular diffusion across the interface due to the onset of crystallization and the growth of spherulites. Spherulite growth requires disentanglement of polymer chains for them to be incorporated in the formed nucleus or growing crystal structure [13]. A loss of entanglements will be detrimental to interlayer bond strength.

### 3.5. Crystalline Morphology at the Weld Interface

**Figure 11:**
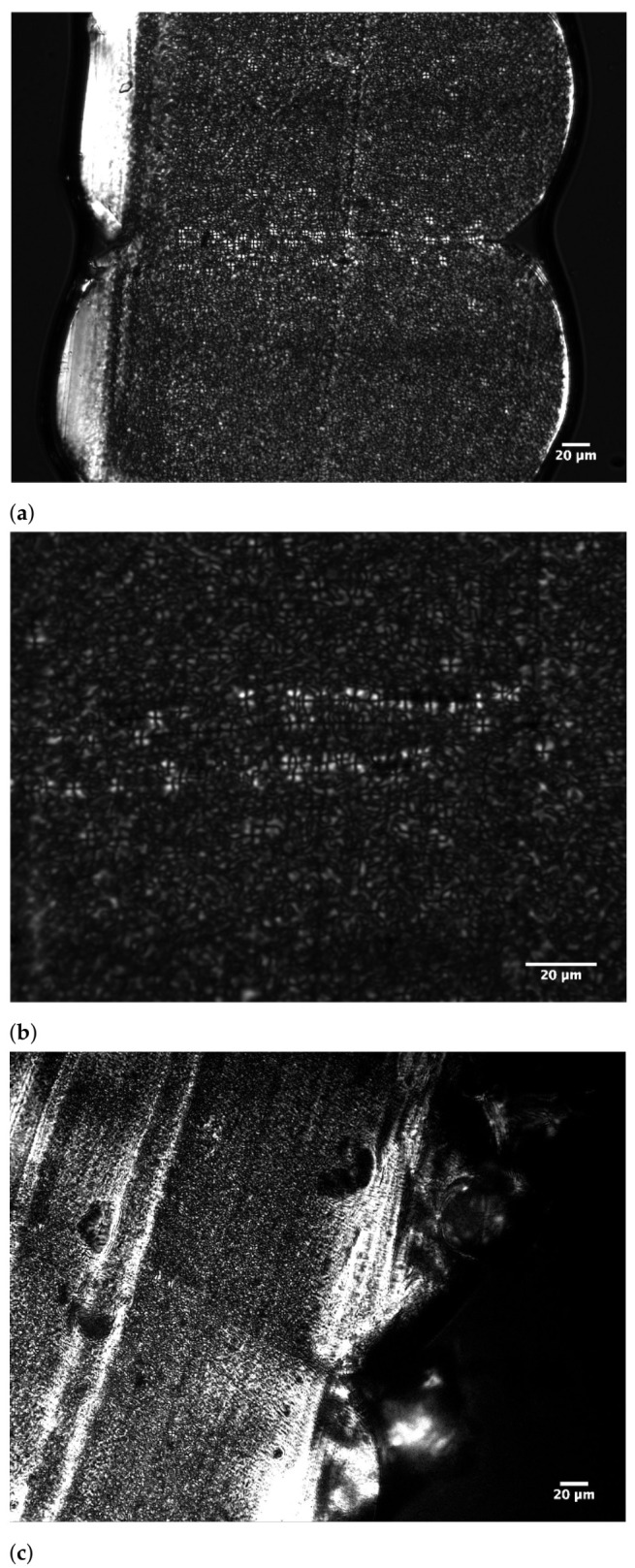

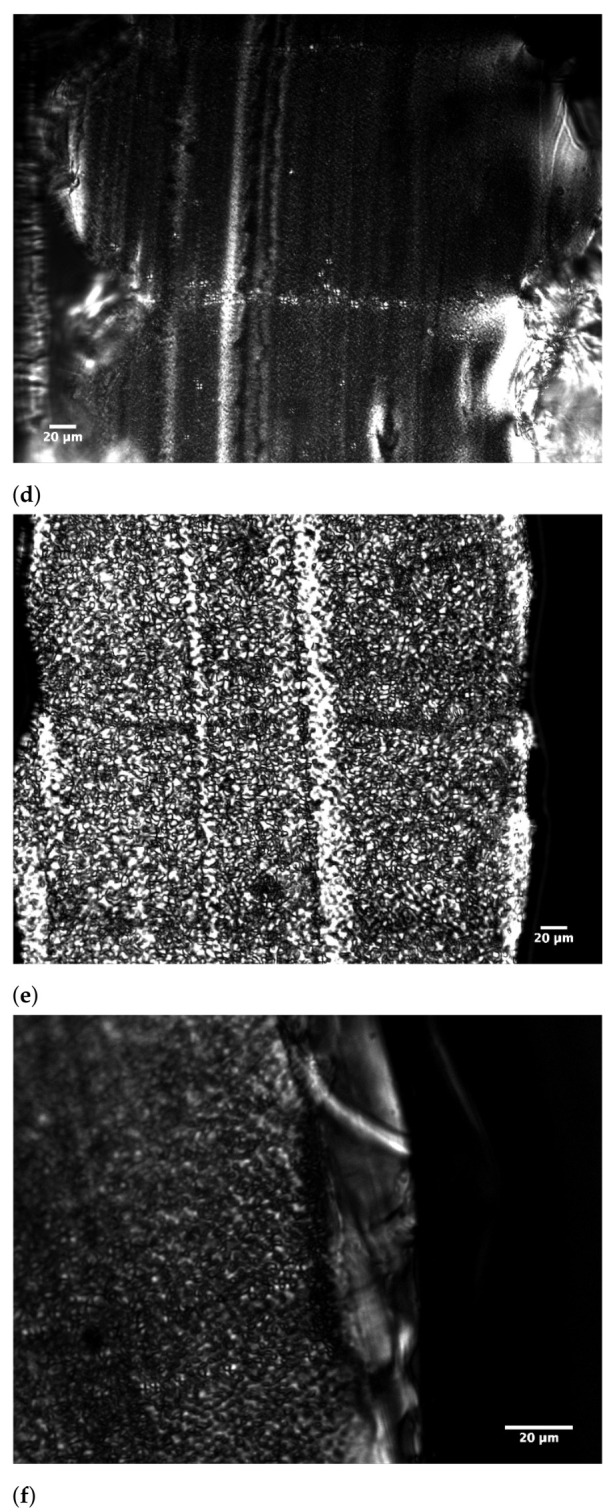
Examples of PLM images depicting the variations in crystalline morphology and spherulite sizes obtained across the distinct printing conditions after FFF processing. (**a**) Condition 1 Layer 10 20×: HMWPA, T_liquefier_ = 260 °C, v_print_ = 11 mm/s and T_build plate_ = 110 °C. (**b**) Condition 1 Layer 10 50×: HMWPA, T_liquefier_ = 260 °C, v_print_ = 11 mm/s and T_build plate_ = 110 °C. (**c**) Condition 2 Layer 40 20×: HMWPA, T_liquefier_ = 260 °C, v_print_ = 11 mm/s and T_build plate_ = 40 °C. (**d**) Condition 8 Layer 40 20×: HMWPA, T_liquefier_ = 260 °C, v_print_ = 5.5 mm/s and T_build plate_ = 40 °C. (**e**) Condition 6 Layer 10 20×: LMWPA, T_liquefier_ = 240 °C, v_print_ = 11 mm/s and T_build plate_ = 110 °C. (**f**) Condition 2 Layer 10 50×: HMWPA, T_liquefier_ = 260 °C, v_print_ = 11 mm/s and T_build plate_ = 40 °C.

**Figure 12:**
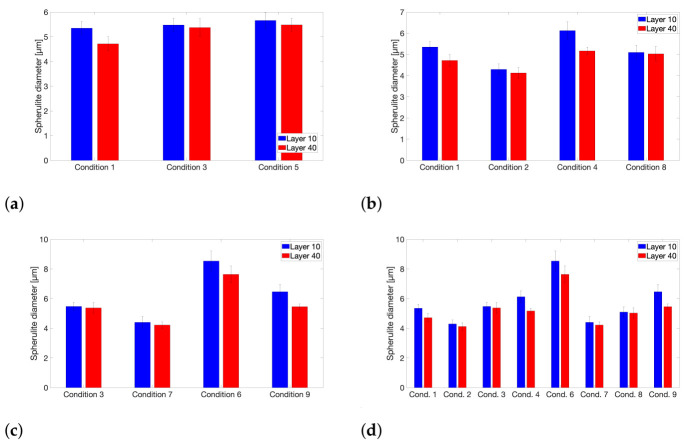
An overview of the measured spherulite diameters for all printing conditions. (**a**) Effect of T_liquefier_ = 260, 240 and 220 °C for conditions 1, 3 and 5, respectively. (**b**) Effect of v_print_ = 11 mm/s for conditions 1 and 2, and 5.5 mm/s for conditions 4 and 8. (**c**) Effect of M_w_. HMWPA for conditions 3 and 7 and LMWPA for conditions 6 and 9. (**d**) Effect of T_build plate_ = 110 °C for conditions 1, 3, 4 and 6, and 40 °C for conditions 2, 7, 8 and 9.

A close examination of the resulting crystalline morphology along the weld interface for each printing condition therefore might be able to hint at a possible explanation for the observed deviation from the weld time prediction in terms of the effect of T_build plate_. Hereto, PLM images are obtained of which some examples are shown in Figure 11 to depict the variations in semi-crystalline microstructure and spherulite size that are developed during FFF printing. Spherulite diameters are measured around each respective interface. The resulting average values for each printing condition and respective layer interface are reported in Figure 12, illustrating the effect of the processing parameters and feedstock molecular weight on the attained spherulite size. Figure 11a,b display the spherulitic morphology at the weld interface between layers 9 and 10 for printing condition 1 at 20× and 50× magnification, respectively. Maltese cross extinction patterns are clearly visible which indicate the formation of spherulites [69]. The spherulites present at the interface exhibit stronger birefringence compared to those in the bulk of the layer, indicating that more perfect spherulites are formed at the weld interface [70]. Yet, spherulite density appears to be greater in the bulk of the layer. This observation could be explained by possible remelting of the top surface of a previously extruded layer, which might have already crystallized to some extent, upon deposition of a new layer, where nucleation at the interface will be facilitated by the presence of remaining nuclei. This can trigger crystallization to occur at higher temperatures, leading to the formation of more perfect spherulites with sharp boundaries [70]. The bulk of the layer will hardly be nucleated and will only start nucleating at lower temperatures at larger undercooling, resulting in less perfect spherulites with a higher level of defects and less intense birefringence, yet a higher spherulite density is achieved since primary nucleation will occur almost simultaneously across the bulk of the layer [13,70].

Concerning the effect of T_liquefier_ on the average spherulite diameters, Figure 12a does not exhibit a clear trend, similarly as for the attained degree of crystallinity, as was already described in Section 3.2. Printing conditions 1, 3 and 5 only differ in terms of the initial peak temperatures that the extruded polymer layers undergo and further experience highly similar thermal histories. Hence, the liquefier temperature seems to not affect the crystallization process during FFF processing. This again explains why the measured weld strength nicely followed the prediction of the extent of interlayer bonding through the equivalent isothermal weld time, which is solely based on thermal history and does not take into account the crystallization phenomenon.

Comparing Figure 11c,d allows to visualize the impact of v_print_ on the crystalline microstructure at the interface, where slightly larger spherulites can be observed for the condition manufactured at lower print speed. Figure 12b provides quantitative proof to further substantiate this claim. Although the effect is rather limited, a slower print speed will indeed promote spherulite growth. This can be attributed to the reduced frequency at which nucleation events occur when printing at slower speeds, since, in this case, more time passes in between each successive temperature peak. Hence, spherulite density will be lowered, yet growth can be improved by the extended build time, as well as the prolonged time that the heated print head will reside over the extruded layer, which will enhance local heat retention [42,64]. It should be noted that for lower build plate temperatures, the print speed did also slightly enhance the attained degree of crystallinity, depicted in Figure 7. However, it is assumed that the impact of v_print_ on both the extent of crystallization and the resulting interfacial morphology will not dramatically affect the interlayer bonding phenomenon as was already illustrated by Figure 10b.

A lower molecular weight will generally result in an enhanced crystallization rate and thus an improved spherulite growth rate [71]. This is reflected by the considerably larger spherulite diameters obtained for the conditions employing the LMWPA feedstock filament as depicted by Figure 12c. Larger spherulites are typically formed when crystallization starts at higher temperatures when a reduced primary nucleation is coupled to enhanced chain mobility for a lower molecular weight [69]. The significant impact of molecular weight on the attained degree of crystallinity, which became apparent from Figure 6, is again a strong indication of the intensified crystallization kinetics for the LMWPA feedstock copolymer. As can be observed from Figure 11e, displaying the PLM image for the interlayer interface between layers 9 and 10 for printing condition 6, a highly crystalline microstructure is obtained. The absence of a clear weld line again corroborates the statement that for this interlayer interface bulk conditions have been reached, which can explain why the trouser tear samples failed across the printed layers instead of along the weld line. Although crystallization and spherulite growth occur to a larger extent for the LMWPA copolymer, molecular mobility and interdiffusion seem to not be dramatically affected by the ongoing crystallization process so that interlayer bonding can sufficiently proceed without being limited by the embedding of chains into growing crystals. Hence, the equivalent isothermal weld time concept could still be utilized to justify the trends in interfacial strength observed among the samples printed with feedstock of distinct molecular weight.

From a direct comparison of Figure 11b,f, depicting the PLM images of the weld region between layers 9 and 10 for conditions 1 and 2, which only differ in terms of build plate temperature, it can be easily recognized that a higher T_build plate_ will induce noticeably larger spherulites. This observation is further affirmed in Figure 12d where the average spherulite diameters at the weld interface for conditions printed at an elevated build plate temperature of 110 °C are compared to their counterparts employing a lower T_build plate_ of 40 °C. Hence, both the attained degree of crystallinity, as well as the spherulite sizes are clearly augmented by an elevated build plate temperature due to crystallization occurring at higher temperatures for a prolonged time [53]. The higher overall temperatures experienced by the printed samples when employing a build temperature of 110 °C will lead to less primary nucleation and a reduced crystal growth rate due to limited undercooling. This leads to larger spherulites of higher crystallinity and with less chain conformational defects that is, entanglements, in the amorphous zones [72]. However, for a lower build plate temperature of 40 °C, the layers cool down quite rapidly to a temperature close to the glass transition temperature. Here, the nucleation rate is high, but the chain mobility is limited. As a result, spherulite sizes will not grow much larger than those of the nuclei from which they originate [69]. Upon crystallization, partial disentanglement of polymer chains is necessary to embed the chains into growing crystals, while remaining entanglement points become excluded from the crystalline regions and become located in the interlamellar amorphous regions [13,70]. Under mechanical loading, these amorphous regions between crystallites are largely responsible for the final mechanical performance, as bridging entanglements and tie chains connecting lamellae are considered to be stress transmitters upon deformation, providing ductility [70,73]. The reduced primary nucleation and crystal growth rate at elevated temperatures, induced by a higher T_build plate_, will lead to larger spherulites of higher crystallinity and a higher level of chain disentanglement. Hence, these samples will exhibit dramatically weaker interlamellar connections and a drastically lower tie chain density, which will lead to more brittle behavior and a weaker interlayer interface as a result. For samples printed at lower T_build plate_, crystallization and the accompanied disentanglement will not proceed to such an extent so that a morphology with more scattered, smaller spherulites is formed where stress is conducted throughout the continuous amorphous region surrounding the spherulites. Due to the associated higher tie chain density, more ductile behavior, reflected in improved interlayer bond strength, is observed [70,72,74].

This could possibly serve as an explanation for the discrepancy between the prediction of interlayer bond strength through the equivalent isothermal weld time and the actual observed tear energies. Purely based on thermal history, the samples printed at elevated build plate temperature should possess improved weld strength, as is reflected by the increased weld time values. However, due to a stronger level of disentanglement and a lower tie chain density occurring at the weld interface induced by crystallization at elevated temperatures over the course of FFF processing at high T_build plate_, interlayer bond strength is negatively impacted, resulting in similar or even poorer interfacial adhesion compared to printing conditions employing a lower build plate temperature.

## 4. Conclusions

As a direct result of an ongoing trend where FFF is evolving from a technique for rapid prototyping to a manufacturing process for the production of functional parts for high-end applications, the necessity to incorporate more engineering and high-performance thermoplastics, which are often semi-crystalline in nature, becomes evident in the current pool of feedstock polymers. However, crystallization can complicate their processing. One of the main drawbacks of the FFF process remains the often poor mechanical performance of the printed parts as a result of insufficient interlayer bonding between successively deposited layers, which can be hindered by the crystallization process due to the incorporation of macromolecular chains into growing crystals, thus drastically limiting molecular mobility. To fully exploit the potential of semi-crystalline feedstock polymers for application with FFF, a better understanding of the impact of crystallization on interlayer weld strength is imperative. Therefore, this study aimed at trying to uncover the relationship between the attained degree of crystallinity after FFF printing, the extent of interlayer bond strength, and the resulting crystalline morphology at the weld interface for two random PA 6/66 copolymers with distinct molecular weights. Special attention is given to the influence of processing parameters, such as T_liquefier_, T_build plate_ and v_print_, as well as the feedstock’s molecular weight on the crystallization behavior and observed interlayer adhesion. As a predictive tool for the extent of interlayer bond strength, the concept of an equivalent isothermal weld time is employed.

The liquefier temperature has been found to not affect the crystallization behavior, as neither the attained degree of crystallinity nor the crystalline morphology is influenced by a change in T_liquefier_. However, a considerably enhanced interlayer bond strength is observed for increased liquefier temperatures, as is also reflected in elevated weld time values, since the peak temperatures experienced by the printed layers will be higher in this case. Hence, the amorphous healing theory can be employed to explain and predict the impact of T_liquefier_ on interlayer adhesion, even for the employed semi-crystalline copolymers. A limited effect of v_print_ on the degree of crystallinity is noticeable when employing a lower T_build plate_. Additionally, slightly larger spherulites are formed during FFF processing at reduced print speed due to a higher local heat retention in the deposited layers. However, no significant change in interlayer bond strength is detected, which was again predicted by the equivalent isothermal weld time. It should be noted that the employed print speeds in this study are rather low and are therefore not expected to induce a substantial change in both the crystallization behavior, as well as the extent of interlayer welding. A lower feedstock molecular weight, on the other hand, is characterized by dramatically enhanced crystallization kinetics apparent from the considerably larger degree of crystallinity and spherulite diameters obtained during FFF printing compared to the HMWPA feedstock counterpart. Although the intensified extent of crystallization for the LMWPA copolymer is expected to negatively impact macromolecular chain mobility, the interlayer interfaces for these samples still possessed the highest weld strength, as was also predicted by the equivalent isothermal weld time. Apparently, sufficient mobility is retained, and interlayer diffusion can occur to ample extent prior to any impact of crystallization on chain mobility. When the build plate is heated to an elevated temperature, crystallization can proceed at higher temperatures, which has been found to considerably increase both the degree of crystallinity, as well as the size of the growing spherulites during FFF processing. Based on thermal history alone, these conditions are expected to lead to improved interlayer diffusion as mobility would be enhanced by the higher overall temperature experienced by the extruded layers. This is also apparent from the higher weld times for these conditions, which predict improved interlayer bonding. Yet, an inverse trend is discovered where interlayer interfaces in samples printed at an elevated T_build plate_ possess similar or even poorer interfacial strength compared to their counterparts processed at a significantly lower build plate temperature. This discrepancy between the predicted extent of interlayer bonding and the actual observed tear energy values can be directly explained by the crystalline morphology at the weld interface. Crystallization occurring at elevated temperatures due to a higher T_build plate_ will lead to a reduced crystal growth rate, inducing a higher crystallinity, a higher level of chain disentanglement and a reduced tie chain density in the interlamellar amorphous regions which dictate the response to mechanical stress. This will lead to poorer mechanical performance and less ductile behavior, reflected in weaker interlayer bond strength.

The direct negative influence of the crystallization phenomenon on the interlayer bond strength attained during FFF processing has therefore been clearly demonstrated by highlighting the significance of the resulting crystalline morphology at the weld zone. Hence, for the analysis of the welding problem of semi-crystalline polymers in FFF, it is important to not only consider the highly non-isothermal temperature profile, which is sufficient when treating amorphous polymers, but additionally the attained degree of crystallinity and especially the crystalline morphology should be taken into account to encompass all aspects that can affect interlayer bonding. The work presented here is thus believed to have contributed to a better understanding of the complicated behavior of semi-crystalline polymers within the context of interlayer adhesion in FFF.

## Figures and Tables

**Table 1:** Material properties of the PA 6/66 copolymers.

Material	M_w_ [kg/mol]	T_g_ [°C]	T_m_ [°C]
HMWPA	58	49	199
LMWPA	24	41	198

**Table 2:** An overview of the examined printing conditions.

Condition	Material	T_liquefier_ [°C]	T_build plate_ [°C]	v_print_ [mm/s]
1	HMWPA	260	110	11
2	HMWPA	260	40	11
3	HMWPA	240	110	11
4	HMWPA	260	110	5.5
5	HMWPA	220	110	11
6	LMWPA	240	110	11
7	HMWPA	240	40	11
8	HMWPA	260	40	5.5
9	LMWPA	240	40	11

**Figure A1:**
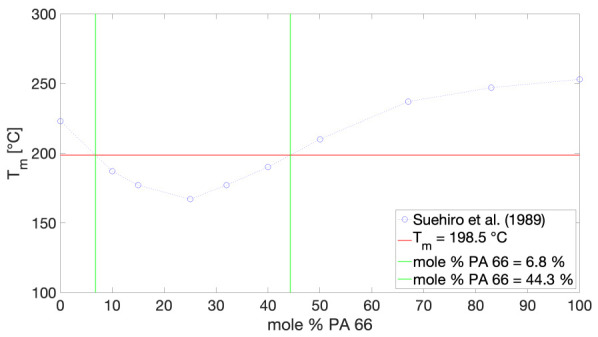
The melting temperature T_m_ versus the molar concentration of PA 66 comonomer (blue dots) for PA 6/66 random copolymers as established by Suehiro et al. (1989) [76]. Both the employed value of the melting temperature (red line) and the two possible PA 66 molar comonomer fractions (green lines) are indicated.

**Figure A2:**
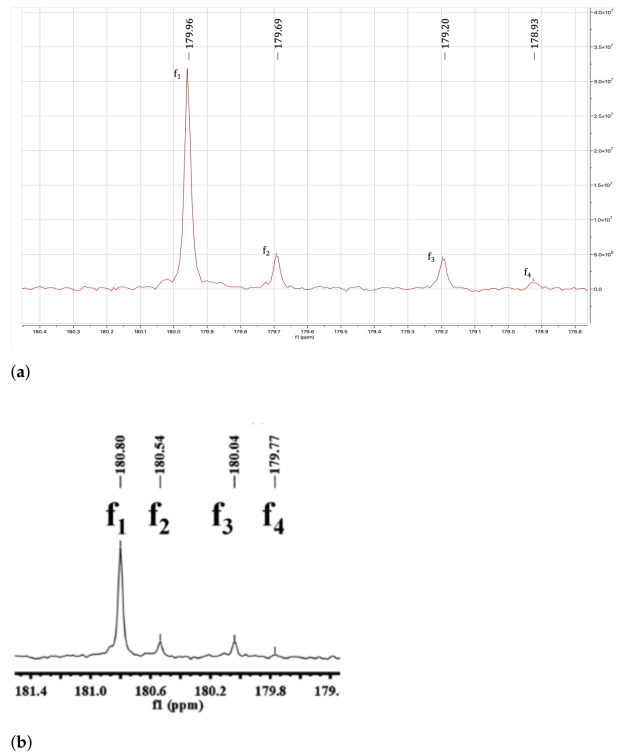
A comparison between the characteristic peaks of the carbonyl groups f_1_ to f_4_ of (**a**) the qualitative ^13^C-NMR spectrum of the employed HMWPA copolymer and (**b**) the quantitative ^13^C-NMR spectrum for a random PA 6/66 copolymer with a PA 66 molar comonomer fraction of 15% as obtained by Tang et al. (2018) [77].

**Figure A3:**
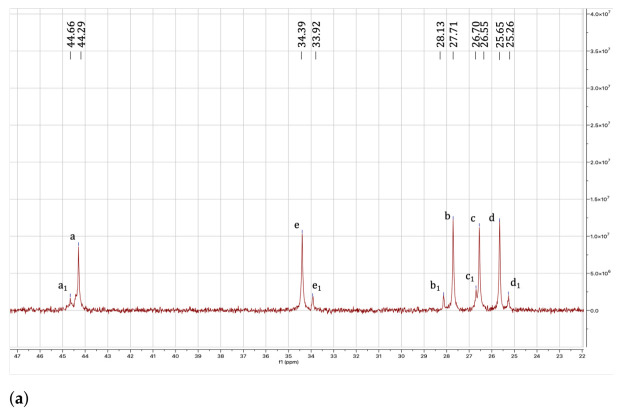

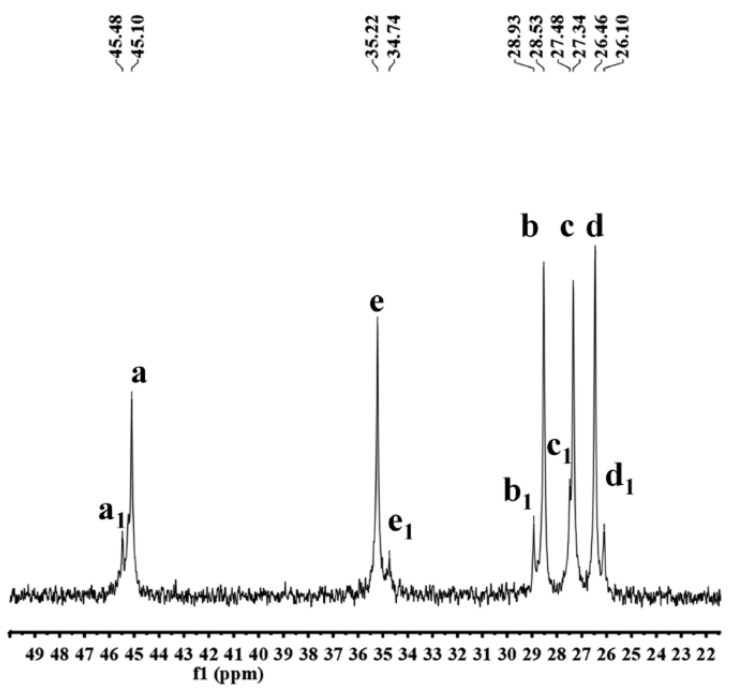
A comparison between the characteristic peaks of the methylene groups a to e and a_1_ to e_1_ of (**a**) the qualitative ^13^C-NMR spectrum of the employed HMWPA copolymer and (**b**) the quantitative ^13^C-NMR spectrum for a random PA 6/66 copolymer with a PA 66 molar comonomer fraction of 15% as obtained by Tang et al. (2018) [77].

**Figure A4:**
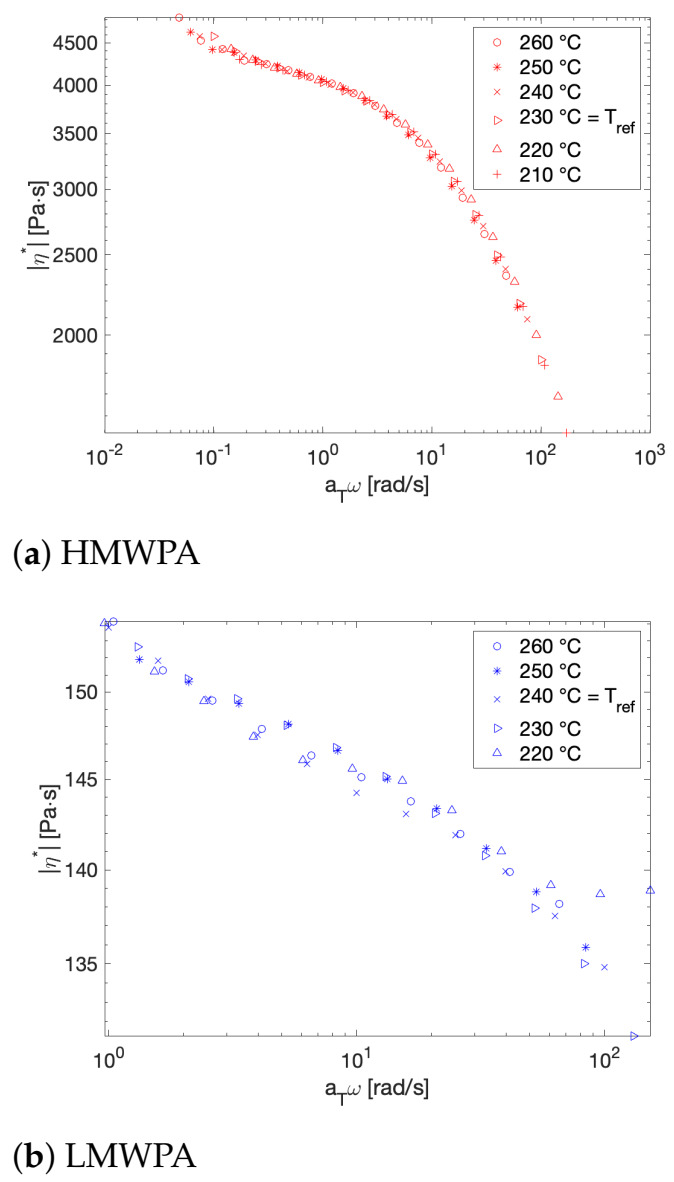
The master curves obtained by applying time-temperature superposition (TTS) on frequency sweep data from rheological measurements in the melt state for (**a**) HMWPA (T_ref_ = 230 °C) and (**b**) LMWPA (T_ref_ = 240 °C).

**Figure A6:**
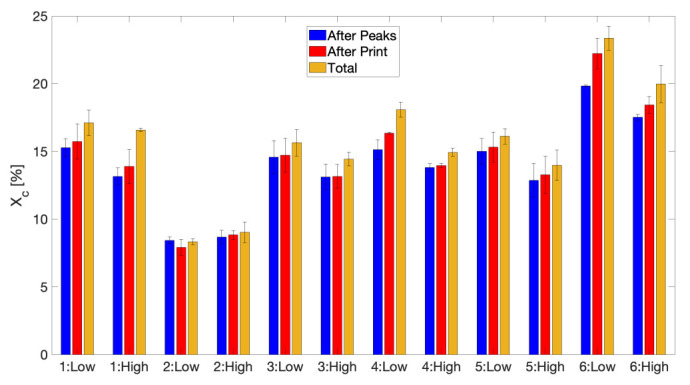
The resulting degrees of crystallinity from FSC measurements for printing conditions 1 to 6 as discussed in [54]. Error bars are added to indicate reproducibility. ‘Low’ and ‘High’ refer to layer 10 and 40, respectively.

**Figure A8:**
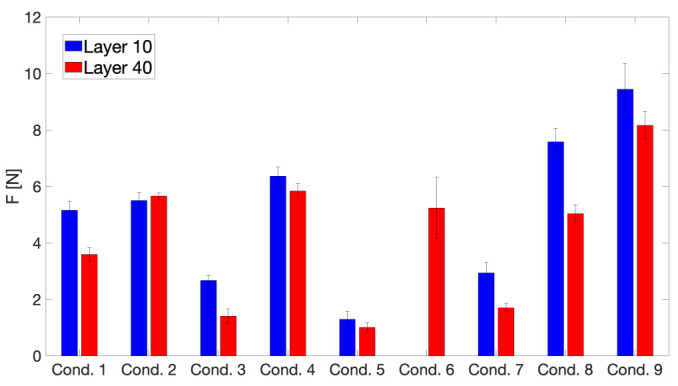
An overview across all printing conditions of the average load for each respective interlayer interface measured through Mode III fracture tests.

**Figure A9:**
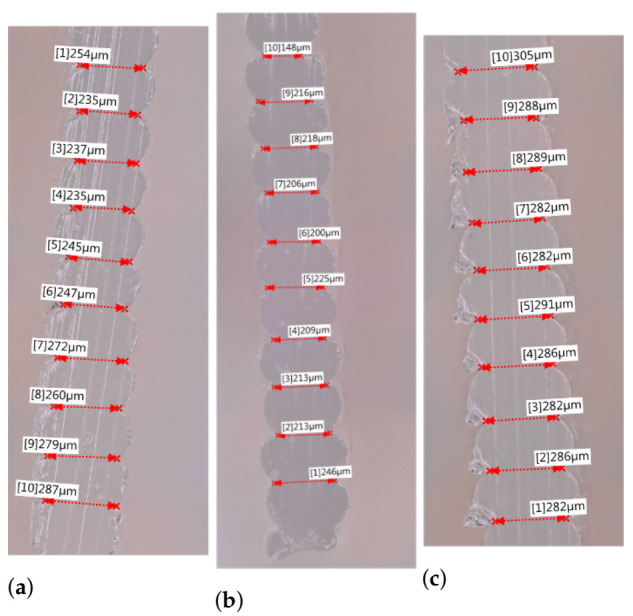
Examples of microscopy images utilized for weld length determination. (**a**) Around layer 10 of Condition 1: HMWPA, T_liquefier_ = 260 °C, v_print_ = 11 mm/s and T_build plate_ = 110 °C. (**b**) Around layer 40 of Condition 3: HMWPA, T_liquefier_ = 240 °C, v_print_ = 11 mm/s and T_build plate_ = 110 °C. (**c**) Around layer 40 of Condition 8: HMWPA, T_liquefier_ = 260 °C, v_print_ = 5.5 mm/s and T_build plate_ = 40 °C.

**Figure A10:**
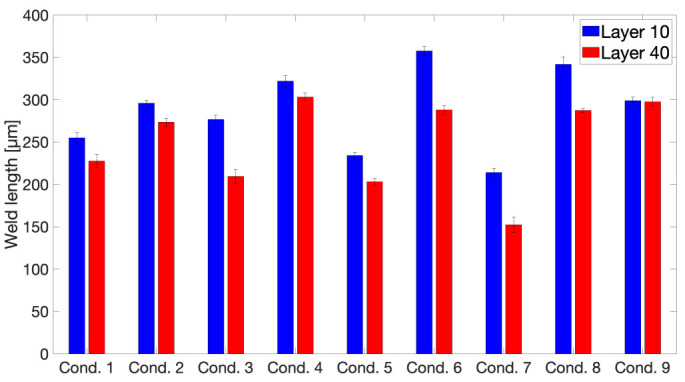
An overview across all printing conditions of the average weld length for each respective interlayer interface measured from microscopy images.

## Data Availability

The data presented in this study are available on request from the corresponding author.

## Appendix A. Determination of the Comonomer Content

To be able to convert the melting enthalpies acquired from FSC to actual degrees of crystallinity, the comonomer content in the utilized copolymer feedstock filaments should be known to discern which comonomer makes up the principal constituent. The melting temperatures of both copolymers are nearly equal. Hence, their comonomer content is considered to be identical, as the comonomer ratio is known to significantly impact the copolymer’s melting point [56,75]. By employing the curve of the melting points of a PA 6/66 random copolymer versus the molar fraction of PA 66 comonomer as established by Suehiro et al. (1989), two possible PA 66 molar fractions can be distinguished [76]. Furthermore, it should be established which of the two comonomers makes up the principal constituent. ^13^C-NMR spectroscopy is applied to acquire an NMR spectrum for the HMWPA copolymer to get insight into the peak positions and relative peak heights corresponding to the signals of the characteristic methylene and carbonyl signals of the PA 6 and PA 66 segments. NMR spectroscopy is performed on a Bruker Avance II+ 600 spectrometer (Bruker Scientific Instruments, Billerica, MA, USA), equipped with a 5 mm PABBO BB (^31^P-^109^Ag)-1H/D probe, at 150 MHz with 30° pulse angle and 1024 transients. The pulse sequence is set as zgig30. The copolymer is dissolved in deuterated sulfuric acid prior to analysis in the spectrometer. As no quantitative NMR spectra could be obtained due to an insufficient signal-to-noise ratio, the NMR spectrum is further analyzed qualitatively through comparison with quantitative ^13^C-NMR spectra acquired by Tang et al. (2018) for random PA 6/66 copolymers with varying PA 66 comonomer fractions [77]. By analyzing the corresponding peak positions and heights and comparing with the spectra from [77], the principal constituent in the copolymers employed in this study can be discerned.

Since comonomer content is assumed to be equal, as the melting temperatures of both copolymers are almost identical, further calculations will be based on an average of both reported melting temperatures that is, 198.5 °C. After all, the resulting comonomer content will not deviate considerably due to a difference in the utilized melting temperature value of 0.5 °C. Based on the melting temperature of 198.5 °C, two distinct values for the molar fraction of PA 66 comonomer in the copolymers can be discerned from Figure A1, which exhibits the change in melting temperature T_m_ for random PA 6/66 copolymers as a function of the mole % of PA 66 comonomer in the random copolymers from the work of Suehiro et al. (1989) [76]. The two possible values for the PA 66 comonomer fraction equal 6.8 mole % and 44.3 mole %, respectively.

It should then be established which of the two obtained values for the PA 66 comonomer fraction is the most likely. Hence, the principal constituent in the employed PA 6/66 random copolymers needs to be determined, which is performed by a comparison between the obtained qualitative ^13^C-NMR spectrum for the HMWPA copolymer and the quantitative spectrum from the work of Tang et al. (2018), where NMR spectra for PA 6/66 random copolymers with varying PA 66 fractions were analyzed [77]. Figure A2 and Figure A3 display a direct comparison between the qualitative ^13^C-NMR spectrum of the employed HMWPA copolymer and the quantitative ^13^C-NMR spectrum as obtained by Tang et al. (2018) for a PA 6/66 random copolymer with a PA 66 comonomer fraction of 15 %, showing the characteristic peaks of the carbonyl groups and methylene groups, respectively. The peak positions for each characteristic peak in the NMR spectra are summarized in Table A1. The reported peak positions of the peaks associated with the carbonyl groups (f_1_ to f_4_) and those associated with the methylene groups (a to e and a_1_ to e_1_) are highly similar indicating that the peaks of the qualitative NMR spectrum of the HMWPA copolymer represent the same carbonyl and methylene groups as those observed by Tang et al. (2018). Peaks f_1_ and f_2_ of the carbonyl groups and a to e of the methylene groups can be attributed to PA 6 segments, while peaks f_3_ and f_4_ and a_1_ to e_1_ correspond to the PA 66 segments [77]. It becomes apparent from Figure A2 and Figure A3 that the heights of the peaks associated with PA 6 segments are much larger relative to those of the peaks corresponding to PA 66 segments. Similar as in the work of Tang et al. (2018) where PA 66 molar fractions up to 15 % were investigated, this relative peak height difference clearly indicates that PA 6 is most definitely the principal constituent in the copolymers employed in this study. The comonomer fraction of PA 66 will thus be appointed a value of 6.8 mole %, equivalent to 93.2 mole % PA 6, which can be converted to 87.3 wt% PA 6 based on the molar masses of both comonomers.

**Table A1:** A comparison between the peak positions of the characteristic peaks for PA 6/66 random copolymers from the ^13^C-NMR spectrum of the employed HMWPA copolymer and the spectrum obtained by Tang et al. (2018) for a PA 6/66 random copolymer with a molar fraction of 15% PA 66 [77].

Peak	Peak Position [ppm]	Peak Position [ppm] from [77]
f_1_	179.96	180.80
f_2_	179.69	180.54
f_3_	179.20	180.04
f_4_	178.93	179.77
a_1_	44.66	45.48
a	44.29	45.10
e	34.39	35.22
e_1_	33.92	34.74
b_1_	28.13	28.93
b	27.71	28.53
c_1_	26.70	27.48
c	26.55	27.34
d	25.65	26.46
d_1_	25.26	26.10

## Appendix B. Rheological Characterization

Rheological characterization of the feedstock copolymers will serve as the basis for the calculation of the equivalent isothermal weld time to determine the temperature dependence of the shift factors. Prior to rheological testing, cylindrical disks are produced from pellets of the employed feedstock copolymers, which are dried for 24 h at 80 °C under vacuum in a a Vacutherm VT 6025 vacuum drying oven (Thermo Fisher Scientific, Waltham, MA, USA). A mold with 25 mm diameter circular openings is filled with the dried pellets and covered with Teflon sheets on either side before being placed in a Collin plate press 200E (Collin, Ebersberg, Germany), which is heated to 220 °C. During compressing molding, the mold is pressed at 220 °C for one minute, followed by two additional minutes at this temperature after a pressure increase of 50 bar. The mold is subsequently cooled to 60 °C after which the disks can be demolded. The resulting compression molded disks are again dried for 24 h at 80 °C before use in rheological measurements. Oscillatory rheological tests are performed on an Ares-2K strain-controlled rheometer (TA Instruments, New Castle, DE, USA) equipped with a forced convection oven which is flushed with inert nitrogen gas. 25 mm diameter parallel plates are utilized, and the gap is set to 1.2 mm. Initially, the linear viscoelastic region is determined for both copolymers by performing strain sweeps. During the strain sweep test, at a constant temperature of 220 °C and a constant angular frequency of 10 rad/s, applied strains vary from 0.1 to 10% with five measurement points per decade. Afterwards, frequency sweeps are performed at a strain within the linear viscoelastic region of each respective copolymer that is, 1% and 10% for HMWPA and LMWPA, respectively. During a frequency sweep, angular frequency ω [rad/s] is varied from 100 to 0.1 rad/s, taking five measurement points per decade. The frequency sweep test is repeated on separate samples for several isothermal temperatures ranging from 210 to 260 °C and 220 to 260 °C for HMWPA and LMWPA, respectively. The resulting data of the modulus of the complex viscosity $|\eta^{*}|$ [Pa·s] obtained as a function of angular frequency for each discrete temperature can then be shifted horizontally relative to a chosen reference temperature to create a single master curve by applying time-temperature superposition (TTS) through the TTS tool of the TA Orchestrator software used to control the employed rheometer. The reference temperatures equal 230 °C and 240 °C for HMWPA and LMWPA, respectively. The resulting master curves are depicted in Figure A4 for both copolymers.

The temperature dependence of the shift factors a_T_ that are thus calculated from TTS can be obtained by fitting to the Williams-Landel-Ferry (WLF) equation, given by Equation (A1). Here, C_1_ and C_2_ are fitting constants, T [°C] is the temperature, and T_ref_ [°C] is the chosen reference temperature. The WLF equation is employed to extrapolate the temperature dependence of the shift factors from 260 °C up until the glass transition temperature for each respective copolymer, although it should be noted that the WLF equation technically is only valid for amorphous polymers within the temperature interval between T_g_ and T_g_ + 100 °C, yet is it utilized anyway due to lack of an adequate alternative [78].

(A1) $$\log a_{T}=\frac{-C_{1}\left(T-T_{ref}\right)}{C_{2}+\left(T-T_{ref}\right)}$$

The fitting constants C_1_ and C_2_ amount to 2.6095 and 210.73 and 2.722 and 295.81 for HMWPA and LMWPA, respectively. As portrayed in Figure A5, the temperature dependence of the shift factors acquired through TTS is extrapolated all the way up until the glass transition temperature for each respective copolymer. When approaching T_g_, the shift factors asymptotically reach infinity illustrating that macromolecular chain mobility ceases completely at T_g_. Since the inverse of the shift factors is utilized as a measure for the level of chain mobility at a specific temperature within the calculation of the equivalent isothermal weld time, it can clearly be seen that temperatures close to T_g_ will hardly contribute to the calculated weld time, while temperatures above the melting point will be mostly responsible for the extent of interlayer bonding. From the inserts in Figure A5, it can be observed that even for temperatures not too far below the melting point, the extrapolated shift factors already have attained considerably higher values, meaning that chain mobility will have been reduced. It should be noted that the improved chain mobility for LMWPA becomes evident from Figure A5, where, when compared at one temperature, the shift factors calculated for LMWPA are much lower than those for the HMWPA copolymer.

## Appendix C. Degree of Crystallinity for Printing Conditions 1 to 6

For printing conditions 1 to 6, an overview of the attained crystalline fractions after each segment in the thermal history mimicked with FSC is provided by Figure A6, where ‘Low’ and ‘High’ refer to layers 10 and 40 in the printed wall geometries. Error bars are added to indicate reproducibility. An extensive discussion of this data can be found in previous work [54].

## Appendix D. Load Data from Fracture Tests

For each printing condition the interlayer bond strength of two separate layer interfaces is studied. The interlayer weld regions under consideration are the interface between layers 9 and 10 and between layers 39 and 40, referred to as Layer 10 and Layer 40, respectively, in all further data representation. Figure A7a depicts a typical example of the raw load F [N] data measured during Mode III fracture tests, in this case of the printed samples for printing condition 1. It can already be noted from Figure A7a that the recorded loads for the weld region between layers 9 and 10 are generally higher than those obtained for the interlayer interface between layers 39 and 40. The displacement range between 20 and 60 mm, which corresponds to the central 20 mm of the interlayer interface, is indicated. The measured load for each fracture test sample is averaged over this displacement interval. For each printing condition and each respective weld region, five fracture test samples are measured, and an average and pooled standard deviation are calculated for each set of five loads averaged over the indicated displacement interval. An overview across all printing conditions of the average load for each respective interlayer interface can be found in Figure A8, where the error bars indicate the calculated pooled standard deviation. For the interlayer interface between layers 9 and 10 of printing condition 6, which utilizes LMWPA as feedstock material and a build plate temperature of 110 °C, no load data is reported. This is illustrated by Figure A7b, where it seems as if the samples failed prematurely during the Mode III fracture tests, so no average load could be calculated. However, the samples for this interlayer interface actually did not fail prematurely along the weld line yet fractured across the printed layers, as is shown in the insert in Figure A7b. This is already a strong indication that the weld region is no longer the weak spot in the printed sample for this specific interlayer interface and printing condition, and it can hence be assumed that a bulk specimen is obtained.

## Appendix E. Weld Length Determination

To take into account variations in contact width between the layers on either side of the interlayer interface, the average loads are normalized by the average weld length for each printing condition and respective layer position to obtain the so-called tear energy G_III_ [kJ/m^2^], as given by Equation (3). Figure A9 illustrates the concept of weld length determination on microscopy images based on two-point manual measurements of the contact widths around the interlayer interface of interest. An overview of the weld length values, calculated as an average of ten measured contact widths around the weld region of interest, across all printing conditions is depicted in Figure A10, where the error bars indicate the standard error on the data.

## References

[1] Turner B.N., Strong R., Gold S.A. (2014). A review of melt extrusion additive manufacturing processes: I. Process design and modeling. Rapid Prototyp. J..

[2] Gao W., Zhang Y., Ramanujan D., Ramani K., Chen Y., Williams C.B., Wang C.C.L., Shin Y.C., Zhang S., Zavattieri P.D. (2015). The status, challenges, and future of additive manufacturing in engineering. CAD Comput. Aided Des..

[3] Ngo T.D., Kashani A., Imbalzano G., Nguyen K.T.Q., Hui D. (2018). Additive manufacturing (3D printing): A review of materials, methods, applications and challenges. Compos. Part B Eng..

[4] Tofail S.A.M., Koumoulos E.P., Bandyopadhyay A., Bose S., O’Donoghue L., Charitidis C. (2018). Additive manufacturing: Scientific and technological challenges, market uptake and opportunities. Mater. Today.

[5] Singh R., Garg H.K. (2016). Fused Deposition Modeling–A State of Art Review and Future Applications. Reference Module in Materials Science and Materials Engineering.

[6] Turner B.N., Gold S.A. (2015). A review of melt extrusion additive manufacturing processes: II. Materials, dimensional accuracy, and surface roughness. Rapid Prototyp. J..

[7] Wohlers T. (2020). Wohlers Report 2020: 3D Printing and Additive Manufacturing Global State of the Industry.

[8] Ligon S.C., Liska R., Stampfl J., Gurr M., Mülhaupt R. (2017). Polymers for 3D Printing and Customized Additive Manufacturing. Chem. Rev..

[9] Peterson A.M. (2019). Review of acrylonitrile butadiene styrene in fused filament fabrication: A plastics engineering-focused perspective. Addit. Manuf..

[10] Rubinstein M., Colby R.H. (2003). Polymer Physics.

[11] Gofman I.V., Yudin V.E., Orell O., Vuorinen J., Grigoriev A.Y., Svetlichnyi V.M. (2013). Influence of the degree of crystallinity on the mechanical and tribological properties of high-performance thermoplastics over a wide range of temperatures: From room temperature up to 250 ^°^C. J. Macromol. Sci. Part B Phys..

[12] Kutz M. (2017). Applied Plastics Engineering Handbook.

[13] Piorkowska E., Rutledge G.C. (2013). Handbook of Polymer Crystallization.

[14] Mark J.E. (2007). Physical Properties of Polymers Handbook.

[15] Li D., Guo G., Fan R., Liang J., Deng X., Luo F., Qian Z. (2013). PLA/F68/Dexamethasone implants prepared by hot-melt extrusion for controlled release of anti-inflammatory drug to implantable medical devices: I. Preparation, characterization and hydrolytic degradation study. Int. J. Pharm..

[16] Patrício T., Domingos M., Gloria A., D’Amora U., Coelho J.F., Bártolo P.J. (2014). Fabrication and characterisation of PCL and PCL/PLA scaffolds for tissue engineering. Rapid Prototyp. J..

[17] Senatov F.S., Niaza K.V., Zadorozhnyy M.Y., Maksimkin A.V., Kaloshkin S.D., Estrin Y.Z. (2016). Mechanical properties and shape memory effect of 3D-printed PLA-based porous scaffolds. J. Mech. Behav. Biomed. Mater..

[18] Singh S., Prakash C., Ramakrishna S. (2019). 3D printing of polyether-ether-ketone for biomedical applications. Eur. Polym. J..

[19] Katschnig M., Arbeiter F., Haar B., van Campe G., Holzer C. (2017). Cranial Polypropylene Implants by Fused Filament Fabrication. Adv. Eng. Mater..

[20] Sacco E., Moon S.K. (2019). Additive manufacturing for space: Status and promises. Int. J. Adv. Manuf. Technol..

[21] Reyes C., Somogyi R., Niu S., Cruz M.A., Yang F., Catenacci M.J., Rhodes C.P., Wiley B.J. (2018). Three-Dimensional Printing of a Complete Lithium Ion Battery with Fused Filament Fabrication. ACS Appl. Energy Mater..

[22] Aslanzadeh S., Saghlatoon H., Honari M.M., Mirzavand R., Montemagno C., Mousavi P. (2018). Investigation on electrical and mechanical properties of 3D printed nylon 6 for RF/microwave electronics applications. Addit. Manuf..

[23] Tichy T., Sefl O., Vesely P., Capal T. Application Possibilities of Fused Filament Fabrication Technology for High-Voltage and Medium-Voltage Insulation Systems. Proceedings of the International Spring Seminar on Electronics Technology.

[24] Gao X., Qi S., Kuang X., Su Y., Li J., Wang D. (2021). Fused filament fabrication of polymer materials: A review of interlayer bond. Addit. Manuf..

[25] Sun Q., Rizvi G.M., Bellehumeur C.T., Gu P. (2008). Effect of processing conditions on the bonding quality of FDM polymer filaments. Rapid Prototyp. J..

[26] Goh G.D., Yap Y.L., Tan H.K.J., Sing S.L., Goh G.L., Yeong W.Y. (2020). Process–Structure–Properties in Polymer Additive Manufacturing via Material Extrusion: A Review. Crit. Rev. Solid State Mater. Sci..

[27] Wang L., Gardner D.J. (2017). Effect of fused layer modeling (FLM) processing parameters on impact strength of cellular polypropylene. Polymer.

[28] McIlroy C., Olmsted P.D. (2017). Disentanglement effects on welding behaviour of polymer melts during the fused-filament-fabrication method for additive manufacturing. Polymer.

[29] Abbott A.C., Tandon G.P., Bradford R.L., Koerner H., Baur J.W. (2018). Process-structure-property effects on ABS bond strength in fused filament fabrication. Addit. Manuf..

[30] Seppala J.E., Hoon Han S., Hillgartner K.E., Davis C.S., Migler K.B. (2017). Weld formation during material extrusion additive manufacturing. Soft Matter.

[31] Bähr F., Westkämper E. (2018). Correlations between Influencing Parameters and Quality Properties of Components Produced by Fused Deposition Modeling. Procedia CIRP.

[32] Davis C.S., Hillgartner K.E., Han S.H., Seppala J.E. (2017). Mechanical strength of welding zones produced by polymer extrusion additive manufacturing. Addit. Manuf..

[33] Aliheidari N., Christ J., Tripuraneni R., Nadimpalli S., Ameli A. (2018). Interlayer adhesion and fracture resistance of polymers printed through melt extrusion additive manufacturing process. Mater. Des..

[34] Bellehumeur C., Li L., Sun Q., Gu P. (2004). Modeling of bond formation between polymer filaments in the fused deposition modeling process. J. Manuf. Process..

[35] Bartolai J., Simpson T.W., Xie R. (2018). Predicting strength of additively manufactured thermoplastic polymer parts produced using material extrusion. Rapid Prototyp. J..

[36] Coogan T.J., Kazmer D.O. (2017). Healing simulation for bond strength prediction of FDM. Rapid Prototyp. J..

[37] Coogan T.J., Kazmer D.O. (2020). Prediction of interlayer strength in material extrusion additive manufacturing. Addit. Manuf..

[38] Mackay M.E. (2018). The importance of rheological behavior in the additive manufacturing technique material extrusion. J. Rheol..

[39] Firas A. (2016). Autohesion of polymers. Polymer.

[40] Jarrousse G. (2005). Self Adhesion of Semi-Crystalline Polymers between Their Glass Transition Temperature and Their Melting Temperature. Ph.D. Thesis.

[41] Barocio Vaca E. (2018). Fusion Bonding of Fiber Reinforced Semi-Crystalline Polymers in Extrusion Deposition Additive Manufacturing. Ph.D. Thesis.

[42] Srinivas V., van Hooy-Corstjens C.S.J., Harings J.A.W. (2018). Correlating molecular and crystallization dynamics to macroscopic fusion and thermodynamic stability in fused deposition modeling; a model study on polylactides. Polymer.

[43] Bhandari S., Lopez-Anido R.A., Gardner D.J. (2019). Enhancing the interlayer tensile strength of 3D printed short carbon fiber reinforced PETG and PLA composites via annealing. Addit. Manuf..

[44] Hertle S., Drexler M., Drummer D. (2016). Additive Manufacturing of Poly(propylene) by Means of Melt Extrusion. Macromol. Mater. Eng..

[45] Xue Y.Q., Tervoort T.A., Rastogi S., Lemstra P.J. (2000). Welding behavior of semicrystalline polymers. 2. Effect of cocrystallization on autoadhesion. Macromolecules.

[46] Li H., Zhang S., Yi Z., Li J., Sun A., Guo J., Xu G. (2017). Bonding quality and fracture analysis of polyamide 12 parts fabricated by fused deposition modeling. Rapid Prototyp. J..

[47] Shmueli Y., Jiang J., Zhou Y., Xue Y., Chang C.C., Yuan G., Satija S.K., Lee S., Nam C.Y., Kim T. (2019). Simultaneous in Situ X-ray Scattering and Infrared Imaging of Polymer Extrusion in Additive Manufacturing. ACS Appl. Polym. Mater..

[48] Spoerk M., Arbeiter F., Cajner H., Sapkota J., Holzer C. (2017). Parametric optimization of intra- and inter-layer strengths in parts produced by extrusion-based additive manufacturing of poly(lactic acid). J. Appl. Polym. Sci..

[49] Qi S., Gao X., Su Y., Dong X., Cavallo D., Wang D. (2021). Correlation between welding behavior and mechanical anisotropy of long chain polyamide 12 manufactured with fused filament fabrication. Polymer.

[50] Costanzo A., Croce U., Spotorno R., Fenni S.E., Cavallo D. (2020). Fused deposition modeling of polyamides: Crystallization and weld formation. Polymers.

[51] Costanzo A., Spotorno R., Candal M.V., Fernández M.M., Müller A.J., Graham R.S., Cavallo D., McIlroy C. (2020). Residual alignment and its effect on weld strength in material-extrusion 3D-printing of polylactic acid. Addit. Manuf..

[52] McIlroy C., Seppala J.E., Kotula A.P. (2019). Combining Modeling and Measurements to Predict Crystal Morphology in Material Extrusion. ACS Symp. Ser..

[53] Wang L., Gramlich W.M., Gardner D.J. (2017). Improving the impact strength of Poly(lactic acid) (PLA) in fused layer modeling (FLM). Polymer.

[54] Vaes D., Coppens M., Goderis B., Zoetelief W., Van Puyvelde P. (2019). Assessment of crystallinity development during fused filament fabrication through Fast Scanning Chip Calorimetry. Appl. Sci..

[55] Wunderlich B. (1971). Macromolecular Physics, Vol. 3, Chap. 8: Crystal Melting.

[56] Telen L., Van Puyvelde P., Goderis B. (2016). Random Copolymers from Polyamide 11 and Polyamide 12 by Reactive Extrusion: Synthesis, Eutectic Phase Behavior, and Polymorphism. Macromolecules.

[57] El Magri A., El Mabrouk K., Vaudreuil S., Chibane H., Touhami M.E. (2020). Optimization of printing parameters for improvement of mechanical and thermal performances of 3D printed poly(ether ether ketone) parts. J. Appl. Polym. Sci..

[58] Lodge T.P. (1999). Reconciliation of the molecular weight dependence of diffusion and viscosity in entangled polymers. Phys. Rev. Lett..

[59] Wolszczak P., Lygas K., Paszko M., Wach R.A. (2018). Heat distribution in material during fused deposition modelling. Rapid Prototyp. J..

[60] Benwood C., Anstey A., Andrzejewski J., Misra M., Mohanty A.K. (2018). Improving the Impact Strength and Heat Resistance of 3D Printed Models: Structure, Property, and Processing Correlationships during Fused Deposition Modeling (FDM) of Poly(Lactic Acid). ACS Omega.

[61] Vanaei H., Shirinbayan M., Deligant M., Raissi K., Fitoussi J., Khelladi S., Tcharkhtchi A. (2020). Influence of process parameters on thermal and mechanical properties of polylactic acid fabricated by fused filament fabrication. Polym. Eng. Sci..

[62] Akhoundi B., Nabipour M., Hajami F., Shakoori D. (2020). An Experimental Study of Nozzle Temperature and Heat Treatment (Annealing) Effects on Mechanical Properties of High-Temperature Polylactic Acid in Fused Deposition Modeling. Polym. Eng. Sci..

[63] Wang L., Sanders J.E., Gardner D.J., Han Y. (2018). Effect of fused deposition modeling process parameters on the mechanical properties of a filled polypropylene. Prog. Addit. Manuf..

[64] Petersmann S., Spoerk-Erdely P., Feuchter M., Wieme T., Arbeiter F., Spoerk M. (2020). Process-induced morphological features in material extrusion-based additive manufacturing of polypropylene. Addit. Manuf..

[65] Gao X., Zhang D., Wen X., Qi S., Su Y., Dong X. (2019). Fused deposition modeling with polyamide 1012. Rapid Prototyp. J..

[66] Levenhagen N.P., Dadmun M.D. (2017). Bimodal molecular weight samples improve the isotropy of 3D printed polymeric samples. Polymer.

[67] Verbeeten W.M.H., Lorenzo-Bañuelos M., Arribas-Subiñas P.J. (2020). Anisotropic rate-dependent mechanical behavior of Poly(Lactic Acid) processed by Material Extrusion Additive Manufacturing. Addit. Manuf..

[68] Wang L., Gardner D.J. (2018). Contribution of printing parameters to the interfacial strength of polylactic acid (PLA) in material extrusion additive manufacturing. Prog. Addit. Manuf..

[69] Peeters M., Goderis B., Vonk C., Reynaers H., Mathot V. (1997). Morphology of homogeneous copolymers of ethene and 1-octene. I. Influence of thermal history on morphology. J. Polym. Sci. Part Polym. Phys..

[70] Huo H., Yao X., Zhang Y., Li J., Shang Y., Jiang S. (2013). In Situ Studies on the Temperature-Related Deformation Behavior of Isotactic Polypropylene Spherulites with Uniaxial Stretching: The Effect of Crystallization Conditions. Polym. Eng. Sci..

[71] Turska E., Gogolewski S. (1971). Study on crystallization of nylon-6 (polycaproamide). II. Effect of molecular weight on isothermal crystallization kinetics. Polymer.

[72] Shang Y., Ning P., Zhang Y., Xue F., Cai Z., Li J., Ma G., Song J., Wu Z., Jiang S. (2018). Study on structure and property relations of *α*-iPP during uniaxial deformation via in situ synchrotron SAXS/WAXS and POM investigations. Polym. Eng. Sci..

[73] McDermott A.G., Deslauriers P.J., Fodor J.S., Jones R.L., Snyder C.R. (2020). Measuring Tie Chains and Trapped Entanglements in Semicrystalline Polymers. Macromolecules.

[74] Thomas C., Seguela R., Detrez F., Miri V., Vanmansart C. (2009). Plastic deformation of spherulitic semi-crystalline polymers: An in situ AFM study of polybutene under tensile drawing. Polymer.

[75] Harvey E.D., Hybart F.J. (1970). Rates of crystallization of copolyamides. II. Random copolymers of nylons 66 and 6. J. Appl. Polym. Sci..

[76] Suehiro K., Egashira T., Imamura K., Nagano Y. (1989). Structural studies on 6-66 and 6-68 copolyamides. Acta Polym..

[77] Tang J., Xu B., Xi Z., Pan X., Zhao L. (2018). Controllable Crystallization Behavior of Nylon-6/66 Copolymers Based on Regulating Sequence Distribution. Ind. Eng. Chem. Res..

[78] Shangguan Y., Chen F., Jia E., Lin Y., Hu J., Zheng Q. (2017). New insight into Time-Temperature correlation for polymer relaxations ranging from secondary relaxation to terminal flow: Application of a Universal and developed WLF equation. Polymers.

